# Luminescent Iridium-Peptide Nucleic Acid Bioconjugate as Photosensitizer for Singlet Oxygen Production toward a Potential Dual Therapeutic Agent

**DOI:** 10.1021/acs.inorgchem.4c05359

**Published:** 2025-03-27

**Authors:** Rosa Maria Dell’Acqua, Veronica Schifano, Maria Vittoria Dozzi, Laura D’Alfonso, Monica Panigati, Paola Rusmini, Margherita Piccolella, Angelo Poletti, Silvia Cauteruccio, Daniela Maggioni

**Affiliations:** a Dipartimento di Chimica, Università degli Studi di Milano, Via Golgi 19, Milano 20133, Italy; b Dipartimento di Fisica “G. Occhialini”, Università degli Studi di Milano-Bicocca, piazza della Scienza 3, Milano 20126, Italy; c Dipartimento di Scienze Farmacologiche e Biomolecolari ″Rodolfo Paoletti″, Dipartimento di Eccellenza 2018-2027, Università degli Studi di Milano, Via Balzaretti 9, Milano 20133, Italy; d Consorzio INSTM, Via G. Giusti 9, Firenze 50121, Italy

## Abstract

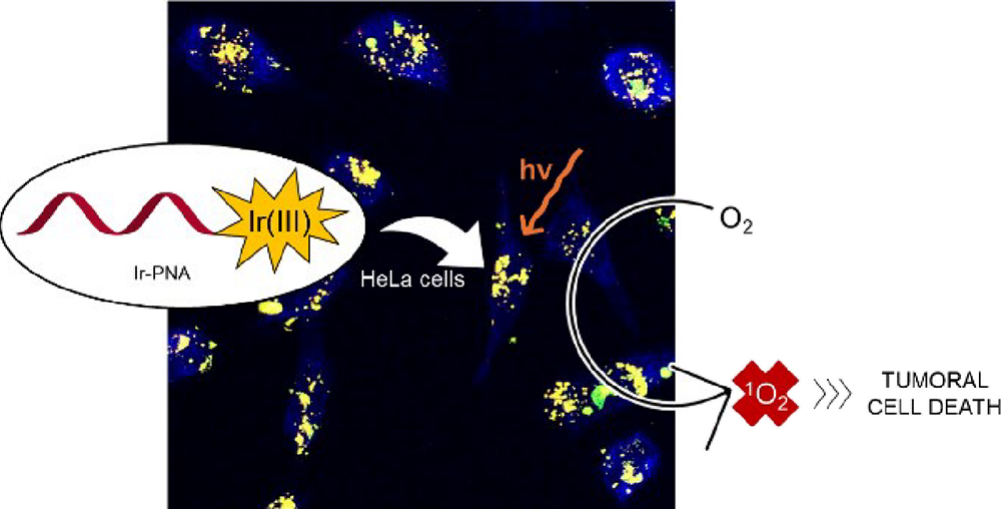

A novel bioorganometallic PNA conjugate (**Ir-PNA**) was synthesized by covalently bonding a model PNA tetramer to a luminescent bis-cyclometalated Ir(III) complex that acted as a photosensitizer under light irradiation to generate singlet oxygen (^1^O_2_). The conjugate was prepared using an Ir complex bearing the 1,10-phenanthroline ligand functionalized with either a free primary amine (**Ir-NH**_**2**_) or a carboxyl group (**Ir-COOH**) for the conjugation to PNA. The photophysical studies on the **Ir-COOH** and the **Ir-PNA** demonstrated that the luminescent properties were maintained after the conjugation of the Ir fragment to PNA. Furthermore, the abilities to produce ^1^O_2_ of **Ir-COOH** and **Ir-PNA** were confirmed in a cuvette under visible light irradiation employing 1,5-dihydroxynaphthalene as a reporter, and the measured singlet oxygen quantum yield (Φ_Δ_) supported the **Ir-PNA** conjugate efficacy as a photosensitizer (Φ_Δ_ = 0.54). Two-photon absorption microscopy on HeLa cells revealed that **Ir-PNA** localized in both the cytosol and nucleus, suggesting its potential as an intracellular carrier for PNA. Cytotoxicity assays by MTT tests showed that **Ir-PNA** was nontoxic in the absence of light, but induced cell death (EC_50_ = 18 μM) after UV irradiation. Overall, the **Ir-PNA** conjugate represents a promising system for the intracellular delivery of the PNA and its application in PDT.

## Abstract 2

A synthetic route for the preparation of a Ir(III)-PNA bioconjugate is proposed. The luminescent compound can be stimulated by two-photon absorption and can enter HeLa cells displaying the formation of vesicle-like structures in all the observed cells and, upon irradiation, produces singlet oxygen causing cell death. Overall, the **Ir-PNA** conjugate represents a promising system for the intracellular delivery of the PNA and its application in PDT.

## 1 Introduction

Photodynamic therapy (PDT) is a noninvasive medical treatment that is able to produce reactive oxygen species (ROS) exploiting light and is categorized into Type I and Type II PDT.^1−3^ The latter consists of exciting molecular oxygen from its triplet ground state (^3^O_2_) to the cytotoxic singlet excited state (^1^O_2_) by using a photosensitizer (PS). First, the PS is excited to the singlet state (S_1_) by suitable light irradiation, followed by intersystem crossing (ISC) to the triplet excited state (T_1_), where its energy is transferred to ^3^O_2_. Conversely, Type I PDT occurs through electron or proton transfer between the PS and the O_2_, leading to the formation of hydroxyl or superoxide radicals.

Among many different PSs,^4^ luminescent organometallic complexes provide several advantages compared to organic fluorophores primarily due to their accessible, low-energy excited triplet states. This feature enables several desirable photophysical properties, including long lifetimes, high photostability, and large Stock shifts, making them highly suitable for biological applications. Additionally, they exhibit minimal background interference, especially if the organometallic compounds can be excited via two-photon absorption (TPA).^5−7^ In this context, different transition metal complexes, especially those containing Ru, Ir, Re, and Pt, are gaining attention as PSs due to their exceptional photophysical, photochemical, and photobiological properties.^8^ In particular, cyclometalated Ir(III) complexes are suitable as PS for ^1^O_2_ production in PDT due to the accessibility of triplet excited states, usually ^3^MLCT.^9^ Many of these compounds possess a positive charge, which promotes their solubility in water, allows interaction with the plasma membrane, and enhances their cellular uptake. Although Ir(III) compounds often exhibit shorter absorption wavelengths compared to their Ru(II) counterparts, they typically show very large Stokes shifts and are much more effectively quenched in the presence of oxygen. This is strictly related to an excellent energy transfer process in Type II PDT, which allows high singlet oxygen quantum yields. In addition, most Ir(III) complexes are characterized by a high two-photon absorption cross section,^7^ making them useful as two-photon PDT agents that can obtain deeper tissue penetration with lower cellular damage. Overall, these unique photophysical features along with their good cell permeability make cyclometalated Ir(III) complexes valuable systems as imaging and sensing probes. Moreover, the conjugation of these complexes to targeting vehicles (i.e., small regulatory peptides)^10^ or to oligonucleotides^11−14^ leads to drug conjugates with enhanced pharmacological properties that find applications in targeted theranostic and antisense technology. In particular, the covalent linkage of Ir(III) polypyridyl complexes to DNA oligonucleotides represents an emerging area for innovative applications of nucleic acids as nanobiosensors for target DNA detection,^12^ and photoredox systems for studying DNA-mediated hole and electron transport.^13,14^

To the best of our knowledge, no examples of cyclometalated Ir(III) complexes conjugated with synthetic mimics of natural nucleic acids have been reported so far, taking into account that chemically modified nucleic acids display improved biological properties compared with natural DNA/RNA (e.g., enzymatic stability, binding affinity) and are valuable tools in therapeutics, clinical diagnostics and nanotechnology.^15^ Among them, peptide nucleic acids (PNAs) belong to the family of xeno-nucleic acids, in which the ribose-phosphate backbone is replaced by the synthetic polyamide-based aminoethylglycine (*aeg*) unit.^16^ This modification allows for a significant increase in the enzymatic stability of PNAs toward nucleases and proteases,^17^ and the neutral *aeg* backbone also ensures strong hybridization properties toward complementary natural nucleic acids, due to the lack of electrostatic repulsion between PNAs and DNA or RNA strands.^18^ However, the great potential of PNAs especially as therapeutic agents is still limited due to their poor cell- or tissue-specific delivery. Diverse approaches have been reported to address the problem of inefficient PNA delivery, including the covalent conjugation of PNA to luminescent organometallic complexes. This represents a useful strategy because it not only enhances the intrinsic PNA properties but also endows PNAs with new additional features depending on the nature of the metal complex.^19^ Examples of PNA oligomers covalently linked to *d*^*6*^ or *d*^*8*^ transition-metal complexes have been reported (e.g., mono^20^ and dinuclear^21^ rhenium(I) complexes, tris(bipyridine)ruthenium(II)^22^ and diamminedichloroplatinum(II)^23^ complexes), and for some of these conjugates the metal complex has proven to be a good carrier for PNA to eukaryotic cells and a nontoxic luminescent tag for PNA labeling. Nevertheless, none of these metal-PNA conjugates have been explored as photosensitizers for PDT, despite some of them having the potential to exhibit all the necessary characteristics for ROS production under appropriate light irradiation. Indeed, very few studies on PNA–PS conjugates have been reported so far, exclusively utilizing organic photosensitizers.^24^ To bridge this gap, this work aims to synthesize and characterize a novel bio-organometallic PNA conjugate, consisting of a model PNA tetramer covalently tethered to a luminescent bis-cyclometalated Ir(III) complex, which can generate singlet oxygen upon light irradiation.

Covalently integrating the emissive Ir(III) complex into the PNA structure could offer several advantages: (i) it enables the tracking of PNA fate in cells otherwise invisible through fluorescence microscopy, as well as through other analytical investigation techniques; (ii) the Ir complex can positively influence the low ability of PNA to cross cell membrane; (iii) the conjugation can create a synergistic effect, combining the therapeutic action of singlet oxygen generated by the Ir complex with the precise genetic targeting capability of the PNA.

**Figure 1:**
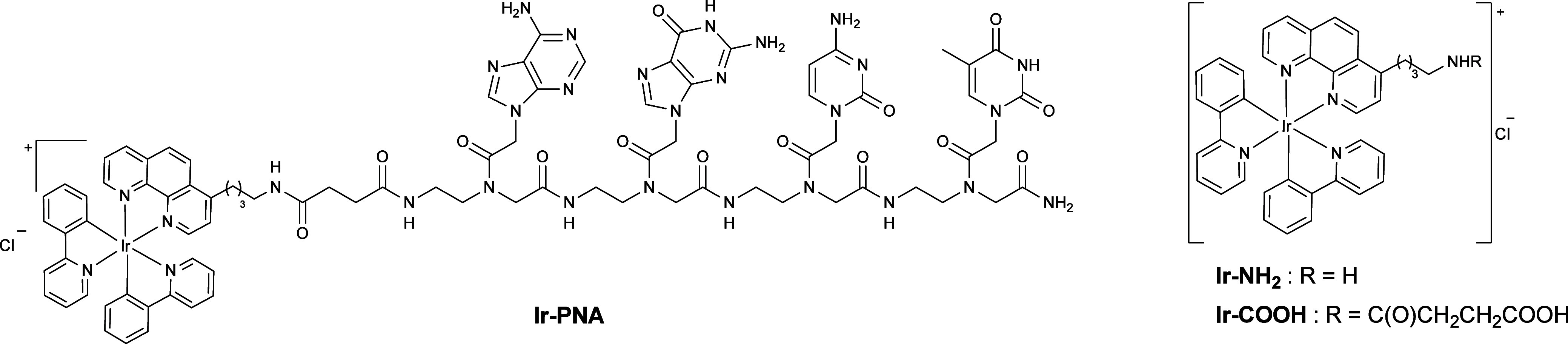
Structure of the **Ir-PNA** conjugate, the **Ir-NH_2_**, and the **Ir-COOH** complexes.

Herein, we report the synthesis of the **Ir-PNA** conjugate (Figure 1), which has been obtained using two different bis-cyclometalated Ir(III) complexes **Ir-NH**_**2**_ and **Ir-COOH** (Figure 1). Both complexes contain, as a third ligand, a 1,10-phenantroline endowed with a proper functional group (i.e., a free primary amine or a carboxylic group) for the covalent conjugation to the model PNA tetramer. The photophysical properties of the novel **Ir-COOH** complex and the **Ir-PNA** conjugate have been fully investigated and exploited to study the uptake and intracellular localization in HeLa cells by confocal microscopy. The capabilities of the **Ir-COOH** and the **Ir-PNA** to act as PSs producing ^1^O_2_ were measured as well, and *in vitro* tests were performed to assess the cytotoxicity in the dark (no irradiation) and after irradiation with UV light.

## 2 Results and Discussion

### 2.1 Design and Synthesis of the Ir-PNA Conjugate

**Scheme 1:**
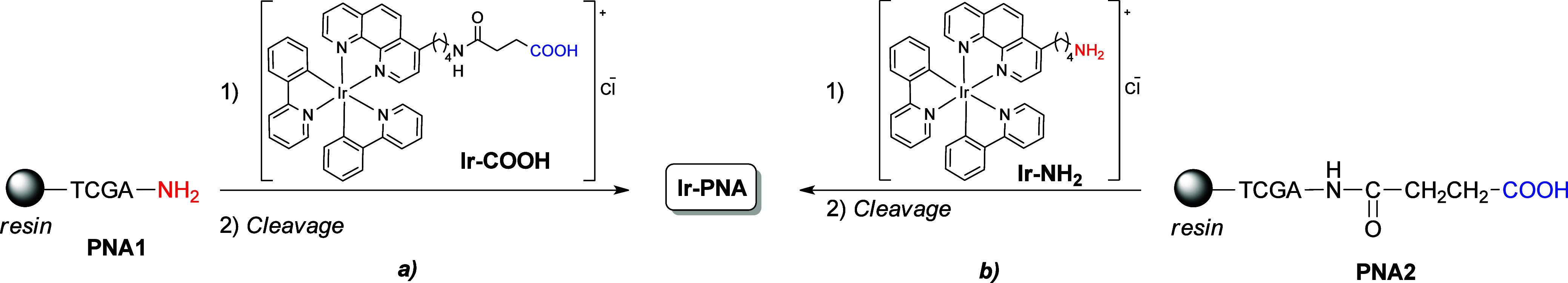
General Strategy for the Synthesis of the **Ir-PNA** Conjugate

Considering the advantages of solid-phase synthesis, the preparation of the **Ir-PNA** conjugate was envisaged through a stepwise solid-phase approach. The Ir complex was conjugated at the *N*-terminal end of the resin-supported PNA tetramer via amide bond formation, followed by the release of the conjugate from the solid support (Scheme 1).

To verify the outcome of the amidation step, two complementary synthetic routes were tested: (a) the amide bond formation between the *N*-terminal end of the **PNA1** with the carboxylic group on the **Ir-COOH** complex (Scheme 1a); (b) the amide bond formation between the carboxylic function in the properly modified **PNA2** and the primary amine group of the **Ir-NH**_**2**_ complex (Scheme 1b).

**Scheme 2:**
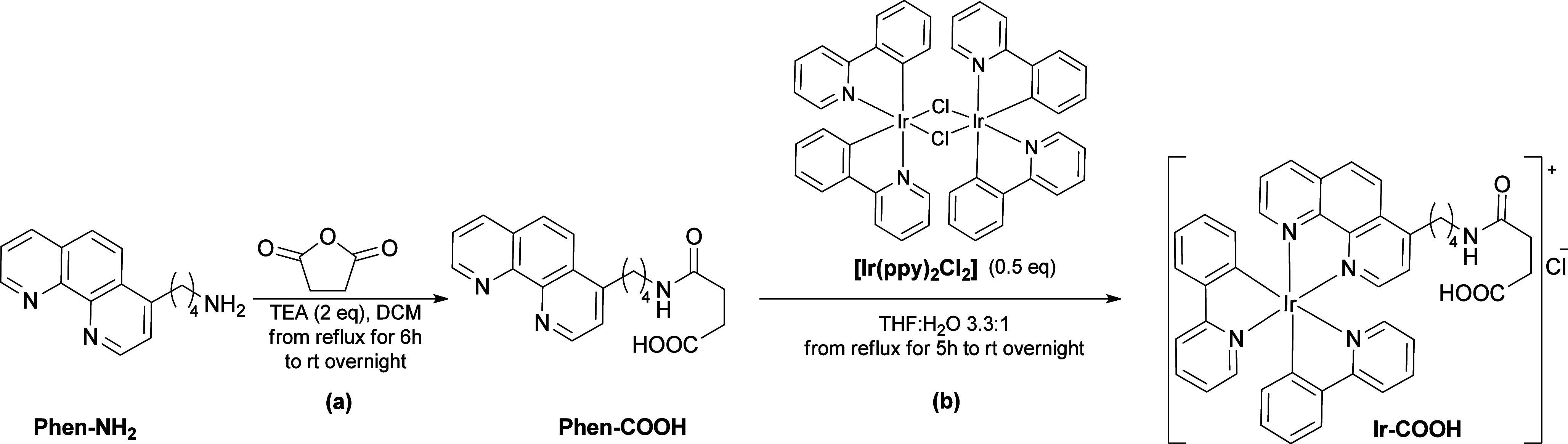
Schematic Procedure for: (a) the Synthesis of the **Phen-COOH** Ligand; (b) the Synthesis of the **Ir-COOH** Complex

The **Ir-NH**_**2**_ complex was prepared according to the literature,^25^ while the **Ir-COOH** complex was synthesized through the reaction between the new ligand **Phen-COOH** with the Ir dimer precursor **[Ir(ppy)**_**2**_**Cl]**_**2**_, following a procedure akin to that used for the synthesis of the **Ir-NH**_**2**_ (Scheme 2).

First of all, the ligand 4-((4-(1,10-phenanthrolin-4-yl)butyl)amino)-4-oxobutanoic acid (**Phen-COOH**) was prepared starting from 4-(1,10-phenanthrolin-4-yl)butan-1-amine (**Phen-NH**_**2**_).^26^ A literature-based procedure was partially followed,^27^ employing triethylamine (TEA) to achieve complete deprotonation of the amine, and utilizing an equimolar amount of succinic anhydride in dry dichloromethane (DCM), resulting in the formation of a terminal carboxylic group (Scheme 2a). The ^1^H NMR spectrum showed a signal pattern consistent with the formation of the desired product (Figure S1). Notably, the amide NH(5′) signal at 7.45 ppm showed a scalar correlation with the methylene (4′) in the α position as observed in a 2D ^1^H COSY experiment (Figure S2). To complete the NMR characterization, both direct and long-range ^1^H–^13^C scalar correlation 2D experiments were acquired and reported in the Supporting Information (Figures S3 and S4).

Next, the **Phen-COOH** ligand underwent a reaction with the Ir dimer precursor **[Ir(ppy)**_**2**_**Cl]**_**2**_ in a mixture of THF and H_2_O, using a method similar to that employed for the synthesis of the **Ir-NH**_**2**_,^25^ but with an increased amount of THF (Scheme 2b), which resulted in a significantly higher yield of 96%. The resulting **Ir-COOH** complex was fully characterized by mono and bidimensional ^1^H and ^13^C NMR experiments (see Figures S5–S10) and electrospray ionization mass spectrometry in positive mode (ESI^+^ MS, Figure S11). NMR spectroscopy allowed ascertaining that the **Ir-COOH** complex was produced as a zwitterion, where the remaining positive charge of Ir(III) is neutralized by the deprotonated carboxylate terminal moiety.

According to Scheme 1, before proceeding with the conjugation of the Ir complex to the PNA tetramer in the solid phase, the stability of the **Ir-NH**_**2**_ was tested under the acidic conditions normally used for the cleavage of PNA from the resin. Hence, the behavior of a solution of **Ir-NH**_**2**_ dissolved in a mixture of trifluoroacetic acid (TFA)/*m*-cresol 9:1 at room temperature was followed over time, monitoring the presence of the MLCT bands, which remained unvaried over 1.5 h (Figure S12). Thus, this test confirmed the stability of this family of Ir complexes, presumably due to the tight chelation of the three ligands, in agreement with the results previously obtained for similar cyclometalated Ir(III) complexes.^10^

Then, the **Ir-COOH** and the **Ir-NH**_**2**_ were conjugated to the PNA starting from the resin-supported, fully protected **PNA1-Fmoc** and **PNA2**, which were prepared according to Fmoc/Bhoc (fluorenylmethyloxycarbonyl/benzhydryloxycarbonyl) manual solid phase synthesis as previously reported.^28^

**Scheme 3:**
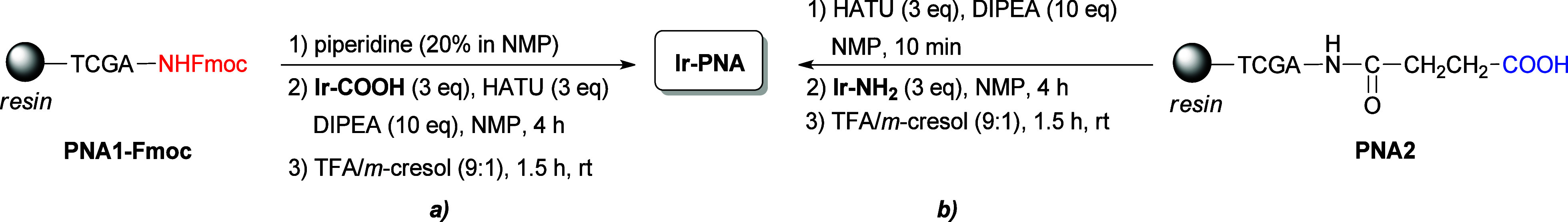
Synthesis of the **Ir-PNA** Conjugate through Two Complementary Routes

The first route to synthesize the **Ir-PNA** conjugate involved the removal of the Fmoc group with a solution of piperidine, followed by the coupling reaction between the free primary amine group of **PNA1** and the carboxylic group of the **Ir-COOH** complex in the presence of *O*-(7-aza-1*H*-benzotriazole-1-yl)-*N,N,N,N*-tetramethyluronium hexafluorophosphate (HATU) as a condensing agent and *N*-ethyldiisopropylamine (DIPEA) as base in *N*-methylpyrrolidone (NMP) for 4 h at room temperature (Scheme 3a).

Next, the treatment of the resin with a solution of TFA/*m*-cresol provided the removal of the *N*-Bhoc protecting groups from the nucleobases and the concomitant cleavage from the resin of **Ir-PNA**, which was purified by reverse-phase high-performance liquid chromatography (RP-HPLC). The **Ir-PNA** identity and purity were confirmed by high-resolution electrospray ionization mass spectrometry (HR-ESI^+^ MS) and RP-HPLC analysis, respectively (Figures S13 and S14).

Alternatively, the resin-supported, fully protected **PNA2** was treated with a solution of HATU and DIPEA in NMP for 8 min and subsequently reacted with a solution of **Ir-NH**_**2**_ in NMP for 4 h at room temperature (Scheme 3b). After the TFA-mediated deprotection of *N*-Bhoc groups and release of the conjugate from the resin, the RP-HPLC trace of the crude **Ir-PNA** was found to be very similar to that obtained following pathway (a), demonstrating the same outcome for both complementary procedures that can be used for the PNA conjugation of this class of bis-cyclometalated Ir(III) complexes.

### 2.2 Photophysical Characterization of Ir-COOH and Ir-PNA

**Figure 2:**
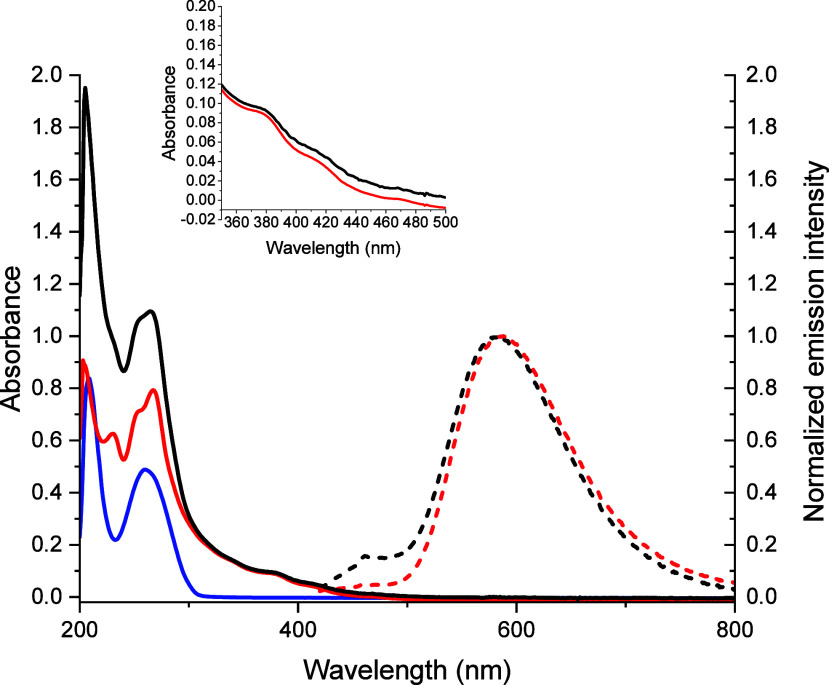
UV–vis absorption (solid lines) and photoluminescence spectra (dashed lines) of the PNA (blue trace), the **Ir-COOH** (red traces) and the **Ir-PNA** (black trace) in methanol at room temperature in aerated conditions. The inset shows a magnification of the MLCT bands centered at 377 and 415 nm.

The photophysical data for the **Ir-COOH** complex and the corresponding **Ir-PNA** conjugate are summarized in Table 1 and depicted in Figure 2 and Figures S15 and S16.

**Table 1:** UV-vis and Photoluminescence Data at Room Temperature for the Compounds **Ir-PNA** (in Aerated Methanol; λ_ex_ = 405 nm), **Ir-COOH** (in Aerated DCM, Acetonitrile, Methanol, and Water; λ_ex_ = 420 nm) and **Ir-NH**_**2**_ (in Aerated DCM, Acetonitrile, and Water; λ_ex_ = 400 nm)

species	solvent	λ_abs_ (nm)	λ_em_ (nm)	Φ	τ (ns)	*k*_r_ (s^–1^)^b^	*k*_nr_ (s^–1^)^b^
**Ir-PNA**	MeOH	377	582	0.039	13.5 (6%)	4.8 × 10^5^	12.0 × 10^6^
		415			80.3 (94%)		
		470					
**Ir-COOH**	CH_2_Cl_2_	381	571	0.084	128.3	6.5 × 10^5^	7.1 × 10^6^
		415					
		472					
	CH_3_CN	374	585	0.027	56.9	4.7 × 10^5^	1.7 × 10^7^
		415					
		470					
	MeOH	377	590	0.034	33.0 (10%)	4.9 × 10^5^	1.4 × 10^7^
		415					
					69.2 (90%)		
		470					
	H_2_O	374	600	0.051	31.8 (3%)	4.5 × 10^5^	8.4 × 10^6^
		412			113.4 (97%)		
		469					
**Ir- NH**_**2**_^a^	CH_2_Cl_2_	383	574	0.052	153	3.4 × 10^5^	6.2 × 10^6^
		417					
	CH_3_CN	376	591	0.017	56	3.1 × 10^5^	1.8 × 10^7^
		414					
	H_2_O	378	604	0.033	2 (2%)	2.8 × 10^5^	8.3 × 10^6^
		417			116 (98%)		

a Literature data from ref (25).

b *k*_r_ and *k*_nr_ indicate the radiative and nonradiative decay constants of the excited states, respectively, and are computed on the most significant lifetime component.

UV–vis absorption spectra of the **Ir-COOH**, acquired in different solvents, display intense absorption bands in the ultraviolet region at about 220–300 nm, whose position is independent of the solvent polarity, and attributable to spin-allowed π-π* ligand-centered ^1^LC transitions. According to the literature data,^29−35^ the bands lying in the range 220–250 nm receive larger contributions from ppy-centered transitions, while those between 250 and 290 nm are mainly centered on the phenanthroline ligand. In the visible region, the **Ir-COOH** complex displays less intense absorption bands in the range 350–420 nm which are mainly attributed to spin-allowed ^1^MLCT [dπ(Ir)-π*(phen)] transition, due to the excitation from filled t_2g_ orbitals of the Ir(III) center to a vacant π* orbital of the phenanthroline ligand, acting as acceptor. However, it should be considered that in Ir(III) cyclometalated compounds, as a consequence of the large covalency of the Ir–C- bond, the HOMO is not completely localized on the d orbitals of the metal center but can be significantly delocalized over the cyclometalating ligands, with a large participation also of the Ir–C- σ bond orbital.^30,31,36^ Therefore, the absorption bands observed in the visible region could be assigned to the σ bond-to-ligand charge transfer (^1^SBLCT) transitions, i.e. the ppy-to-phen charge transfer (^1^LLCT) transitions (see Figure S15). Finally, the absorption bands at the lowest energy (at about 470 nm) are assigned to spin-forbidden CT transitions, which steal the intensity from the spin-allowed transitions thanks to the large spin–orbit coupling induced by the heavy iridium center. The CT character of these absorption bands is confirmed by the observed solvatochromic effect, affording a progressive blue shift on going from the least polar (DCM) to the most polar (water) solvent used in these measurements (see Table 1 and Figure S15). All of these features were expected and showed to be in line with the many orthometalated mononuclear Ir(III) complexes of the [Ir(N^C)_2_(N^N)]^+^ family described in the literature.

The conjugation of the Ir complex to the PNA generates only small modifications of the absorption spectrum in the ultraviolet range, affording a new band at 210 nm due to the PNA backbone transitions and an increase of the absorption at 260 nm due to the ^1^LC transitions of the purine bases (Figure 2). Moreover, as expected, the visible part of these absorption spectra is not modified either by the presence of the PNA oligomer in the **Ir-PNA** or by the free primary amine instead of the carboxylic group in **Ir-NH**_**2**_ (see Figure 2 and Table 1).^25^ Indeed, the presence of the aliphatic chain hampers any electronic conjugation between the PNA, or the functional groups (COOH and NH_2_), and the 1,10-phenanthroline ligand involved in the electronic transitions.

Upon excitation at 405 nm, the **Ir-COOH** and **Ir-PNA** display photoluminescence emission bands in the yellow-orange range of the visible spectrum, attributable to a metal-to-ligand charge transfer transition having triplet character (^3^MLCT) (see Figure S16). The broad and structureless shape of these emission bands suggests the absence of the role of the π-π* LC transitions in the emission process, further confirmed by the excitation spectra (Figure S17) and the monoexponential decay profile of the lifetimes observed in DCM and acetonitrile (ACN) solutions. The slight blue shift of the emission band observed in the DCM solution for **Ir-COOH** and **Ir-NH**_**2**_, with respect to the analogous Ir complex containing the unsubstituted 1,10-phenanthroline ligand (Ir-Phen, λ_em_ = 582 nm in DCM),^33^ reflects the weak electron donor ability of the aliphatic substituent on the phenanthroline ligand, thus confirming the charge transfer character of the emission.^30,33,35^ Moreover, both **Ir-COOH** and **Ir-NH**_**2**_ display a red-shift of the emission maximum passing from the least polar (DCM) to the most polar (water) solvent,^37^ in agreement with the commonly observed trend of excited states having charge transfer character (see Table 1). The red shift of the emission maximum is usually associated with a progressive decrease of the photoluminescent quantum yield (PLQY) and a shortening of lifetimes, in agreement with the energy gap law.^38^ Nevertheless, it is interesting to note that in the case of **Ir-COOH** and **Ir-NH**_**2**_, the lifetimes and PLQYs do not follow this trend. As for the **Ir-NH**_**2**_ complex,^25^ also **Ir-COOH** displays higher PLQY in polar solvents such as water or methanol than in the less polar solvent acetonitrile, together with longer and biexponential lifetimes (see Table 1). This behavior suggests that, in these polar solvents, the **Ir-COOH** is also present as a nanosized aggregate, as already observed for the previously reported **Ir-NH**_**2**_ complex, where the complex may be affected by a more rigid and less polar surrounding. To confirm this hypothesis, we measured the photophysical properties of **Ir-COOH** dissolved in ACN mixed with increasing quantities of H_2_O (see Figure S18 and Table S1). As the water content increases, a progressive red-shift of the emission maximum is observed, followed by an increase in fluorescence quantum yield and a corresponding lengthening of lifetimes, which become progressively biexponential (Figure S19). This behavior supports the hypothesis that, in highly polar solvents, the emission is mainly governed by nanoaggregates. The quantity of these nanoaggregates increases with higher water content, although they do not account for the entire amount of the compound present in the solution. It is to be noted that the slight shortening of the main lifetime component observed in neat water—corresponding to a 2-fold increase in the *k*_*nr*_ constant (see Table S1)—strongly suggests that water acts as a classical quencher, capable of competing with nonradiative deactivation processes, as previously reported for other dyes.^39^

The progressive formation of nanoaggregates was confirmed by DLS measurements (Figure S20). In neat acetonitrile and H_2_O/ACN mixtures with ratios of 1:9, scattering signals were either absent or insufficient to be detected. However, on increasing the water content, an increase in scattered photons was observed and, parallelly, the fitting of the correlation functions returned dimensional distributions with larger populations. Also for the MeOH solution of **Ir-COOH**, DLS measurements confirmed the presence of a population centered at 300 nm, alongside a numerically prevailing population attributable to the single molecules in solution (Figure S21).

It is important to note that nanoaggregates larger than 50 nm are undetectable by ^1^H NMR spectroscopy. Accordingly, the ^1^H NMR spectrum of the **Ir-COOH** complex in methanol-*d*_4_ (Figure S5) displayed only sharp signals, which are characteristic of the nonaggregated molecular fraction, providing only a partial view of the overall system.

The formation of these nanoaggregates seems to be further favored by the presence of the hydrophobic PNA chain in the **Ir-PNA** conjugate, thus accounting for an 8 nm blue shift of the emission maximum in water and the further increase in PLQY together with the main component of the lifetime. Indeed, the short component (τ1 = 33.0 and 13.5 ns for the **Ir-COOH** and the **Ir-PNA**, respectively), observed in the biexponential emission decay kinetics, can be attributed to the free molecules (which are more affected by the presence of oxygen and more exposed to the polar solvent), while the longer component (τ2 = 69.2 and 80.3 ns for the **Ir-COOH** and the **Ir-PNA**, respectively), is attributed to the nanoaggregates.

### 2.3 Photochemical Stability of the Ir-COOH and the Ir-PNA

To evaluate the durability of the **Ir-PNA** conjugate under extended exposure to light in the presence of oxygen, we conducted a photochemical stability test. This assessment was needed as a critical requirement for the potential use of **Ir-PNA** as a photosensitizer. Hence, the compound was dissolved in methanol, and the solution was saturated with O_2_, bubbling the gas through the solution for 5 min. Subsequently, the solution was subjected to visible light exposure, using a monochromatic LED light (λ = 400 nm, 15.2 mW/cm^2^) and UV–vis absorption spectra were recorded every few minutes for a total period of irradiation of 20 min (Figure S22). Analysis of the superimposed absorption spectra obtained at various time points demonstrated the high photostability of the compound. The same stability test was replicated for the **Ir-COOH** complex, yielding similar outcomes (Figure S23).

### 2.4 Photoreaction of the Ir-COOH and the Ir-PNA with 1,5-Dihydroxynaphtalene as an Indirect Reporter of Singlet Oxygen Production

Previously, the ability of the **Ir-NH**_**2**_ complex to serve as a PS inducing the generation of singlet oxygen was evaluated.^25^ This assessment was based on the use of the singlet oxygen indirect reporter 1,5-dihydroxynaphthalene (DHN).^34^ The same photoreaction assay was conducted in this work, under irradiation with monochromatic LED light (λ = 400 nm, 15.2 mW/cm^2^) within a cuvette for both **Ir-COOH** and **Ir-PNA** complexes, to compare their performances as PS using the estimation of their singlet oxygen quantum yields Φ_Δ_, which are reported in Table 2.

**Table 2:** Estimated Quantum Yields of Singlet Oxygen Production (φ_Δ_) and Kinetic Constant (*k*_obs_) of the Studied Compounds in Methanol

species	*k*_obs_ (min^–1^)	ν (10^–6^ M min^–1^)	I_λ=400_ (10^–7^ mol min^–1^)	Φ_Δ_
**Ir(ppy)**_**3**_	0.0401	6.82	7.00	0.50^a^
**Ir-PNA**	0.0158	2.60	2.48	0.54
**Ir-COOH**	0.0146	2.44	2.83	0.44

a From ref (34).

**Scheme 4:**
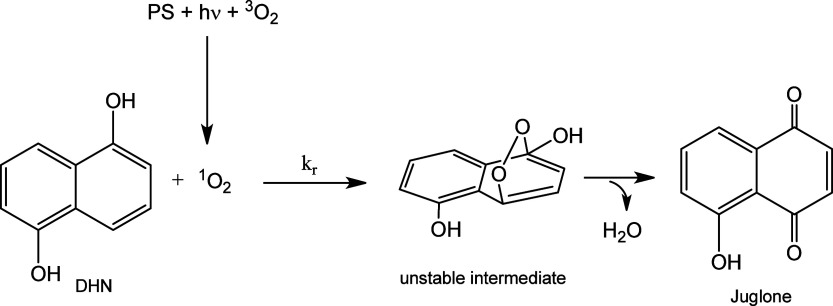
Depiction of the Photochemical Conversion of DHN in Juglone

A schematization of the selective and quantitative photoreaction of DHN with ^1^O_2_ is reported in Scheme 4.

The PS is excited to a singlet excited state upon irradiation with sufficiently energetic photons; then, it undergoes intersystem crossing leading to the population of a triplet excited state, and finally, through energy transfer, the PS promotes the excitation of molecular oxygen from its triplet ground state to the singlet cytotoxic excited state. The resulting photoproduced ^1^O_2_, in the presence of an excess of DHN, undergoes a quantitative reaction as illustrated in Scheme 4, ultimately forming the oxidized species Juglone.

**Figure 3:**
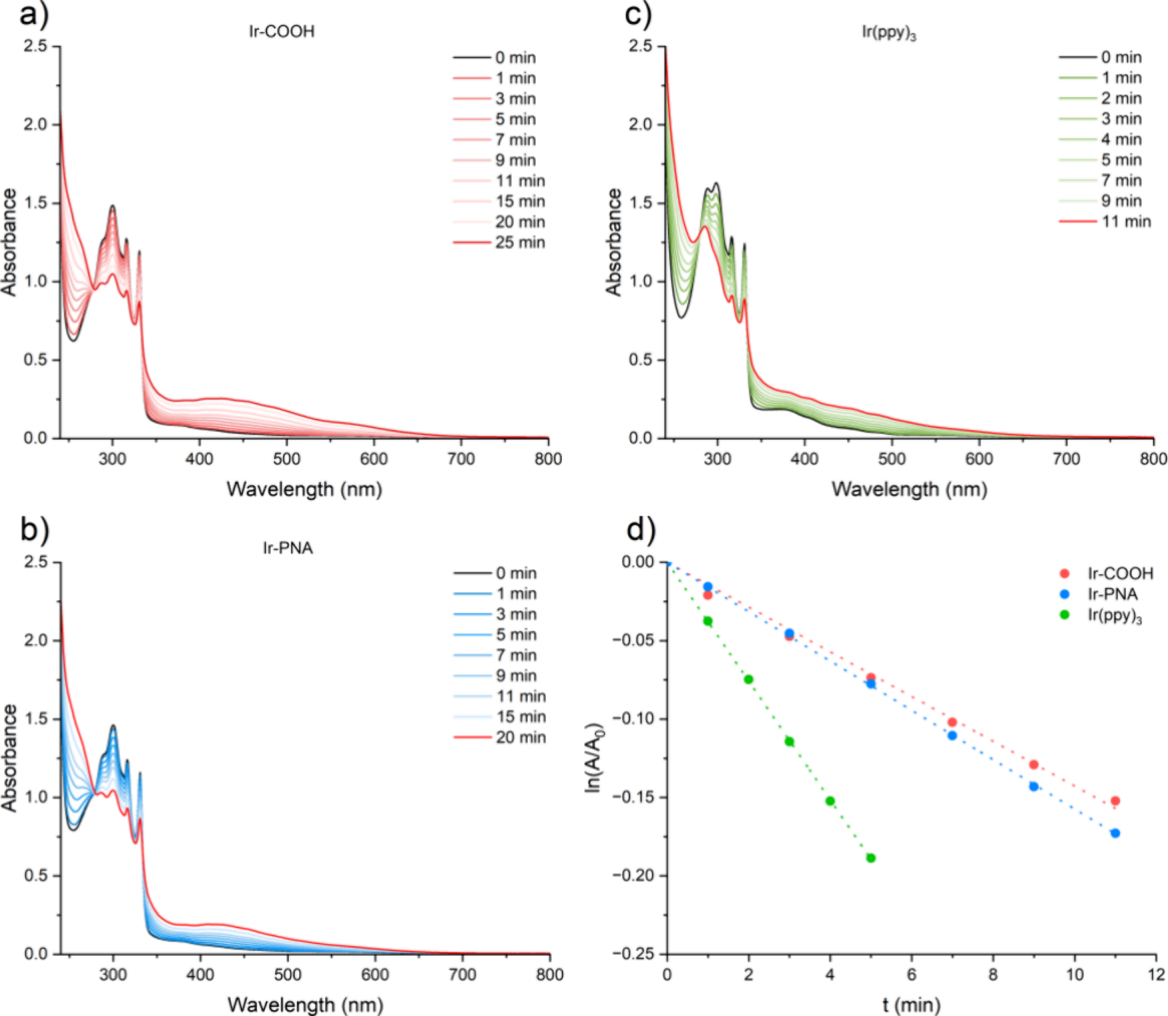
Evolution of UV–vis absorption spectra of DMC/methanol 9:1 solutions containing DHN (a) **Ir-COOH** (1.27 × 10^–5^ M), (b) **Ir-PNA** (1.12 × 10^–5^ M), (c) **Ir(ppy)_3_** (1.18 × 10^–5^ M) irradiated for several minutes (λ = 400 nm). (d) Comparison of the semilogarithmic plots of DHN consumption as a function of the irradiation time registered for the investigated sensitizers, with *A*_*t*_ and *A*_0_ referring to the typical DHN absorbance value (at 316 nm), registered at irradiation times *t* and 0, respectively.

The comparison of Φ_Δ_ determined for the **Ir-COOH** complex (0.44) and the **Ir-PNA** conjugate (0.54) shows that, in these conditions, ^1^O_2_ is generated even more efficiently after the conjugation of the Ir complex to the PNA chain. This suggests that the PS properties of the Ir complex are neither compromised nor affected by its conjugation with the PNA tetramer.

### 2.5 Fluorescence Microscopy Localization Study by Two-Photon Absorption

**Figure 4:**
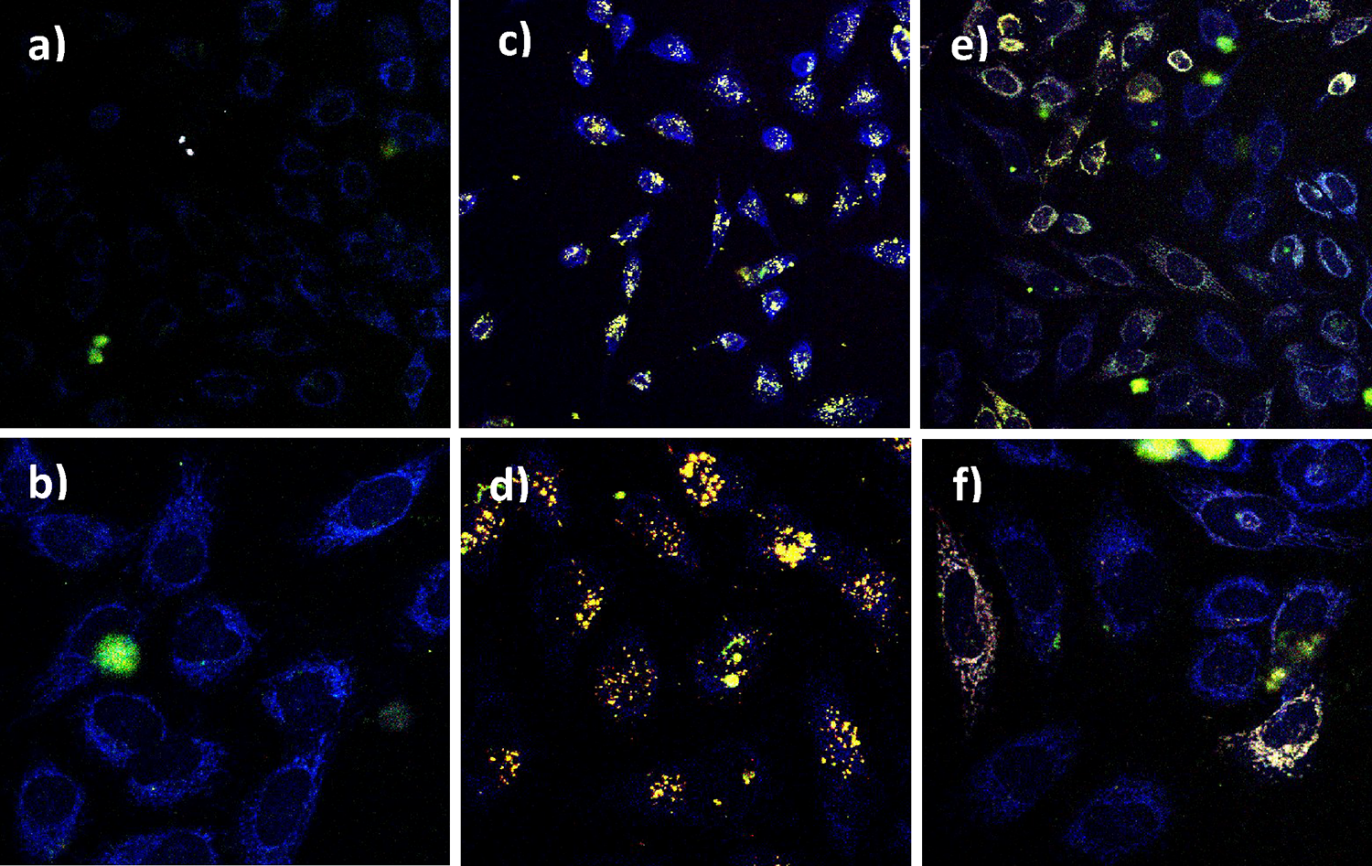
TPE microscopy images at two distinct magnifications (a, c, and e = 100×, 300 × 300 μm^2^ field of view; b, d, and f = 50×, 150 × 150 μm^2^ field of view) of vehicle-treated HeLa cells (control, panels a and b), HeLa cells treated with the **Ir-PNA** (panels c and d) and HeLa cells treated with the **Ir-COOH** (panels e and f) for 21 h. The z-scan images (panels d and f) are the superposition of several images acquired along the optical axis at 1 μm intervals.

Cellular uptake and intracellular localization in HeLa cells of both **Ir-COOH** and **Ir-PNA** compounds were monitored by two-photon-excitation (TPE) microscopy. HeLa cells were treated with 25 μM of each compound and incubated for 21 h. After this incubation period, the cells were observed under the microscope. Notably, TPE at 720 nm was utilized to excite the iridium compounds and acquire microscopy images (Figure 4) as described in the Experimental Part. The control exhibited a slight autofluorescence (Figure 4a,b) detectable in the blue channel (480 ± 30 nm), predominantly attributed to NADH (λ_em_ = 460 nm), as documented in previous literature for TPE excitation.^40^ Both the **Ir-PNA** and the **Ir-COOH** compounds (Figure 4c–f) displayed luminescence in the green and red channels (535 ± 50 and 600 ± 40 nm, respectively), consistent with their broad emission spectra (Figure 2). Images captured at the highest magnification (Figure 4d and 4f) were acquired through z-scan techniques to confirm that the signal originated from within the cell rather than the outer membrane. Clearly, the emission resulting from the treatment with the **Ir-PNA** conjugate originated from all visible cells within the observed frame. In contrast, with the **Ir-COOH** complex, emission predominantly arose from a pool of treated HeLa cells. This discrepancy implies that the internalization process may be more facilitated for the PNA adduct compared to the neutral form of the **Ir-COOH** complex, which is primarily present at physiological pH (being deprotonated, the terminal COOH group balances the residual positive charge of the Ir(III) metal center). Moreover, apart from the positive charge retained by the **Ir-PNA** conjugate, this has a higher lipophilic character, which could also enhance its internalization. Nevertheless, the **Ir-PNA** conjugate appeared to form aggregate-like spots, probably due to its limited solubility in water, mainly accumulating in the cytosol as well as in the nuclei, where a green/red emission is observable. On the contrary, the **Ir-COOH** complex showed, where present, a more homogeneous distribution at the cytoplasm level without any emission coming from the nuclei. Further studies will be dedicated in the near future to better characterize the molecular mechanisms through which the **Ir-PNA** conjugate is internalized in cells, and colocalization studies will also be carried out.

### 2.6 PDT Treatment on HeLa Cells

**Figure 5:**
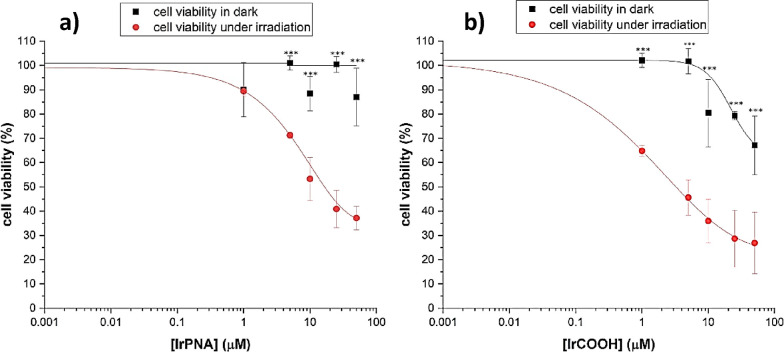
Dose–response plots for the **Ir-PNA** conjugate (panel a) and the **Ir-COOH** complex (panel b) in the dark (black curves) and under UV irradiation. One-way ANOVA corrected for multiple comparison by Sidak’s post hoc analysis was performed, ***p < 0.001; *n* = 4.

The effectiveness of the **Ir-COOH** complex alone and its conjugate with PNA in inducing photocytotoxicity and cytotoxicity in the dark were assessed and compared employing HeLa cells. The cells were treated separately with increasing doses of both compounds either in the dark or under light exposure. HeLa cells were incubated for 24 h to promote internalization, as suggested by the previous TPE microscopy observations (see paragraph 2.5). Potential dark cytotoxicity was assessed by using an MTT assay. The results, presented in Figure 5, indicate that the **Ir-COOH** complex induced significant dark cytotoxicity, in a dose-dependent manner. On the other hand, cells treated with the **Ir-PNA** conjugate maintained high cell viability (>90%) in the dark across all the tested concentrations. This characteristic is crucial for a photosensitizer to be considered for further *in vivo* applications.

This level of toxicity for the iridium complexes was already observed in previous studies conducted on HeLa cells, where the similar complex **Ir-NH**_**2**_ displayed higher cytotoxicity in the dark compared to the corresponding conjugate with an amide-based polymer.^25^

Concurrently, the same experiment was performed by irradiating the samples for 30 min, using a low-intense UV- lamp emitted by a polychromatic irradiation source (5.2 mW/cm^2^ in the 200–800 nm range, see emission profile in Figure S24). To avoid any overheating due to the lamp exposure, the temperature during the irradiation time was constantly monitored and maintained at 37 °C. The dose–response curves for the **Ir-COOH** and the **Ir-PNA** compounds (shown in Figure 5, panels (a) and (b), respectively) were fitted nonlinearly using a sigmoidal function to determine dark and light EC_50_ values. These values represent the effective concentration required to reduce cell viability to 50% in the dark and after irradiation (Table 3). Both compounds showed higher cytotoxicity upon UV light exposure. However, the light EC_50_ was 1 order of magnitude higher for the **Ir-PNA** conjugate compared to the **Ir-COOH** complex, indicating greater phototoxicity when the Ir complex was not bound to the PNA. Considering the insights gained from TPE microscopy images, the lower photocytotoxicity of the **Ir-PNA** conjugate could be attributed to its tendency to form aggregates, confined in vesicle-like structures (see Figure 4). These aggregates might potentially trap singlet oxygen produced during irradiation and hinder it from reaching target biomolecules effectively. On the other hand, the amplified cytotoxicity showed by **Ir-COOH** could be possibly due to the additional contributions of the intrinsic toxicity in the dark.

**Table 3:** Cytotoxicity of the **Ir-PNA** and **Ir-COOH** Compounds on Hela Cells in the Dark and upon Irradiation, Expressed in Terms of Dark EC_50_ and Light EC_50_

species	dark EC_50_	light EC_50_	PI^a^
**Ir-PNA**	>50 μM	18 μM	>2.8
**Ir-COOH**	>50 μM	3.5 μM	>14.3

a PI: phototherapeutic index = dark-EC_50_/light-EC_50_.

Additionally, the lower limit phototherapeutic indexes (PI) were calculated from the dark to light EC_50_ values (see Table 3), reflecting the amplification of cytotoxic activity by light treatment. Our complexes exhibit low phototherapeutic indexes if compared to other orthometalated Ir complexes of the same [Ir(N^C)_2_(N^N)]^+^ family,^41,7^ which show PI even 1 order of magnitude higher than ours. This could be due to many different factors, such as cellular uptake as well as subcellular localization within a particularly sensitive subcellular organelle. Nevertheless, making a fully reliable comparison is challenging, as certain variables should be standardized, such as the administered light dose, the incubation time both before and after irradiation, and, of course, provided that the comparison of different molecular effects is conducted within the same cell line. Our data are preliminary, and a complete biological study has not been carried out yet.

## 3 Conclusions

To the best of our knowledge, this study marks the first instance of a conjugate between an iridium complex and a PNA tetramer, and although the literature contains notable examples of studies on PNA-metal complex conjugates, none have exploited these conjugates for PDT applications. The conjugation did not compromise the emissive properties of the iridium complex or its ability to produce singlet oxygen. On the contrary, the iridium complex bound to the PNA, in a cuvette under a nonpolar environment (DCM/MeOH 9:1), showed a higher singlet oxygen yield Φ_Δ_ (0.54) compared to the one of **Ir-COOH** (0.44). Furthermore, the singlet oxygen generated under irradiation did not appear to damage the PNA tetramer, as confirmed by the UV–vis spectra of irradiated **Ir-PNA**, which remained unchanged.

More importantly, biological tests on HeLa cells demonstrated that the **Ir-COOH** complex enters cells more efficiently when conjugated to PNA, as all of the cells in the TPE microscope images exhibited emission in the green/red channels. In contrast, the free **Ir-COOH** complex was not internalized in a significant portion of the cells, which displayed only autofluorescence in the blue channel. This reduced cellular uptake may be due to a combination of lower lipophilicity and a neutral net charge of the **Ir-COOH** complex, which at pH > 2–3 exists as a zwitterion (with the deprotonated carboxyl group balancing the residual +1 net charge of the Ir(III) bis-orthometalated ion). In comparison, the **Ir-PNA** conjugate shows enhanced lipophilicity due to the presence of the tetramer and retains the residual positive net charge of the metal ion, which is not counterbalanced in the adduct. Nonetheless, starting from 25 μM dose on, the **Ir-COOH** complex exhibited increased cytotoxicity in the dark compared to the **Ir-PNA**, which, on the contrary, did not cause cellular death under the same conditions. This level of toxicity for the iridium complexes was already observed in previous studies conducted on HeLa cells. When irradiated, both Ir-based compounds showed an increase in cytotoxicity, more pronounced for **Ir-COOH,** possibly due to the additional contributions of the intrinsic toxicity in the dark and its light-triggered cytotoxicity. Conversely, the **Ir-PNA** remained nontoxic in the dark and exhibited cytotoxicity only upon irradiation.

Overall, the Ir complex appears to transfer its beneficial properties to the conjugate by helping PNA to enter cells, while the PNA seems to mitigate the negative characteristics of the complex in terms of dark cytotoxicity.

Based on these results, our next step will be to select an appropriate PNA sequence capable of acting as an antisense agent, and after conjugating it with the **Ir-COOH** complex, we will test the corresponding Ir-PNA conjugate on different cell lines to study the potential synergistic effect in inducing the death of diseased cells of the conjugate with respect to the two separate components.

## 4 Experimental Section

### 4.1 Synthesis of the Phen-COOH Ligand and the Ir(III) Complexes Ir-NH_2_ and Ir-COOH

Commercial reagents were used without further purification. 4-(4′-Aminobutyl)-1,10-Phenanthroline (**Phen-NH**_**2**_) was synthesized as previously described.^26^**[Ir(ppy)**_**2**_**Cl]**_**2**_ was prepared using a published method with IrCl_3_·3H_2_O as a precursor (BASF).^42^ All the manipulations for the syntheses of both **Phen-NH**_**2**_, **[Ir(ppy)**_**2**_**Cl]**_**2**_ and **Ir-NH**_**2**_ were performed under nitrogen using oven-dried Schlenk-type glassware. THF was distilled from sodium/benzophenone just before its use. NMR experiments were acquired on a Bruker DRX400 spectrometer equipped with a Bruker 5 mm BBI Z-gradient probe head with a maximum gradient strength of 53.5 G/cm (π/2 pulse: ^1^H 8.5 μs, ^13^C 13 μs) operating at 400.13 and 100.62 MHz for ^1^H and ^13^C NMR, respectively. The ESI^+^ MS analyses were recorded with a Thermo Fisher LCQ Fleet ion trap mass spectrometer. Elemental C, H, and N analyses were performed on a PerkinElmer CHN 2400 instrument.

### 4.2 Solid Phase Synthesis of the Ir-PNA Conjugate

The resin-supported **PNA1-Fmoc** and **PNA2** were prepared by solid phase Fmoc/Bhoc synthesis, as previously described.^28^ The protected PNA monomers (Fmoc-PNA-T–OH; Fmoc-PNA-C(Bhoc)–OH; Fmoc-PNA-G(Bhoc)–OH; and Fmoc-PNA-A(Bhoc)–OH) were purchased from ASM Research Chemicals GmbH (Hannover, Germany). The H-rink amide *ChemMatrix* was purchased from Merck. Polypropylene one-way syringes (1.5 or 4 mL) and corresponding PTFE frits, used as reaction vessels, were purchased from SepaChrom (Milan, Italy). Solid phase syntheses were performed by using an orbital rotator shaker at 500 rpm. Centrifugation steps were performed using a 0.6 LISA centrifuge. The RP-HPLC purification of the **Ir-PNA** conjugate was performed on an Agilent 1200 Series system, using the semipreparative column Luna C18 (250 × 10 mm, 5 μm) at a flow rate of 3 mL/min. Solvent A (0.1% TFA in water) and solvent B (0.1% TFA in acetonitrile) were used in the following gradient: 0% B (5 min), B 0–50% (30 min), B 50–100% (1 min), B 100% (5 min), B 100–0% (1 min), and B 0% (3 min). This method was used with detection by UV (220, 260, 280, 360, and 366 nm). The RP-HPLC analyses of the purified **Ir-PNA** were performed using the analytical column Luna C18 (150 × 4.6 mm, 5 μm) at a flow rate of 1 mL/min, using the same gradient reported for the semipreparative column. High-resolution electrospray ionization mass spectrometry (HR-ESI^+^ MS) analyses were acquired in a positive polarity with a Synapt G2-Si QToF instrument (Waters) interfaced through a ZsprayTM ESI-probe for electrospray ionization (Waters). Data were processed with a MassLynxTM v4.2 software (Waters). The concentrations of **Ir-PNA** solutions were determined by measuring the absorbance at 260 nm with an Agilent 8453 UV/vis spectrophotometer. The molar extinction coefficient of the PNA tetramer was calculated according to standard protocol.^43^

### 4.3 Photophysical Characterization

UV–vis absorption spectra were acquired on a single beam Agilent model 8543 spectrophotometer equipped with a diode array detector using a 1 cm path length quartz cuvette at room temperature. Molar absorption coefficients of MLCT transitions were measured by weighting a known amount of Ir complex and quantitatively dissolving it in a known MeOH volume. The stock solution was then used to prepare diluted solutions at different molar concentrations (in the range 6 × 10^–5^–1.7 × 10^–4^ M), which were employed to acquire UV–vis absorption spectra, from which the absorbance values at 377 and 415 nm were collected. The slopes of the straight lines obtained by plotting the absorbances vs molarity returned the ε (M^–1^ cm^–1^). The estimated experimental errors are 2 nm on the absorption maxima and 5% on the molar absorption coefficients. Emission spectra were acquired by using an Edinburgh FLS980 spectrofluorimeter equipped with a 450 W xenon arc lamp. Emission spectra were corrected for source intensity (lamp and grating) and emission spectral response (detector and grating) by standard correction curves. The estimated experimental errors are 2 nm on the PL bands maxima. Time-resolved measurements were performed using the time-correlated single-photon counting (TCSPC) option on the FLS980. The pulsed excitation source, an LED at 404 nm, was mounted directly on the sample chamber, and the emission was collected by a multichannel plate MCP-PMT Hamamatsu H10720–01 single photon-counting detector. The photons collected at the detector were correlated by a time-to-amplitude converter (TAC) to the excitation pulse. The data analysis was performed using the commercially available F980 software (Edinburgh Instruments). The goodness of the data fitting was assessed by minimizing the reduced chi-squared function (χ^2^). The uncertainty on fluorescence lifetimes is estimated to be ±0.05 ns.

Photoluminescence quantum yields (Φ) were collected for an optically diluted solution (<10^–5^ M) using wavelength scanning with a Hamamatsu C11347–11 Quantaurus-QY absolute PL quantum yield spectrometer, equipped with a xenon light source (150 W), a monochromator, and a Spectralon integrating sphere, and employing the commercially available U6039–05 PLQY measurement software (Hamamatsu Photonics Ltd., Shizuoka, Japan). The photoluminescence quantum yields were measured by exciting the samples between 350 and 430 nm.

### 4.4 Two-Photon Excitation Microscopy Localization Study

Two-photon excitation (TPE) fluorescence microscopy experiments were conducted using a 720 nm excitation beam generated by a mode-locked Ti:sapphire laser (Mai Tai HP, Spectra Physics). The laser emits pulses with a duration of 120 fs full width at half-maximum and a repetition frequency of 80 MHz, delivering 20 mW of power at the sample plane. The optical setup comprises a confocal scanning head (M610 scanning mirrors module, ISS) integrated onto an upright optical microscope (BX51, Olympus), equipped with a high working distance objective (XLPlan, NA = 1.05, wd = 2 mm, 25x water immersion, Olympus). Fluorescence signals, collected in epifluorescence geometry by the same objective, were directed to a nondescanned detection unit and then transmitted to three Hamamatsu analog output photomultipliers (HC125–02, Hamamatsu). Sample emissions were filtered using 480/30, 535/50, and 600/40 band-pass filters to eliminate scattering contributions. Fluorescence data were processed by using the ISS Vista Vision Suite software (ISS). The microscopy images presented in this paper are the result of 4 averaged scans, with a residence time of 10 μs per pixel. The field of view for the 512 × 512 pixel images ranged from 300 to 50 μm^2^, depending on the zoom factor employed. Z-scan images were acquired at 1 μm intervals.

### 4.5 Photochemical Stability Study

The photochemical stability test and photoreaction with DHN in the presence of investigated systems were monitored by employing a Jasco V-650 spectrophotometer. The experiments were performed by directly irradiating a 3 mL quartz cuvette with a monochromatic LED light with a maximum emission centered at 400 nm (BRIDGELUX, USA). The average full emission intensity reaching the cuvette corresponded to ca. 15.2 mW/cm^2^, as regularly checked with a Thorlabs PM200 optical power meter equipped with a thermal Thorlabs S302C power sensor. The normalized emission spectrum of the LED is reported in Figure S25.

### 4.6 Biological Study

For the MTT tests, formazan formation was measured spectrophotometrically at 570 nm using the microplate reader (PerkinElmer, EnSpire, Waltham, MA, USA). The phototoxicity tests on HeLa cells were carried out by using a low-intensity and mainly UV-light-emitting polychromatic irradiation source, with an average intensity emission of ca. 5.2 mW/cm^2^ in the 200–800 nm range. The normalized emission spectrum of the employed irradiation source is reported in Figure S24.

### 4.7 Synthesis of the [Ir(ppy)_2_(Phen-NH_2_)]Cl Complex (Ir-NH_2_)

[Ir(ppy)_2_(Phen-NH_2_)]Cl was prepared by slightly varying a literature procedure.^25^ [Ir(ppy)_2_Cl]_2_ (88.6 mg, 0.0827 mmol) was suspended in 20 mL of freshly distilled THF, obtaining a yellow suspension in which 40.8 mg of **Phen-NH**_**2**_ (0.163 mmol) was added. After the addition of 10 mL of H_2_O Milli-Q, the brown solid aggregates of **Phen-NH**_**2**_ were completely dissolved. The mixture was then heated at 70 °C under an inert atmosphere, and after a few minutes, the cloudy yellow suspension turned completely clear. Once the set temperature was reached, it turned emerald-green, denoting a slight defect of ligand. After heating the mixture for 5 h, a nominal slight excess of **Phen-NH**_**2**_ (3.2 mg, 0.0127 mmol) was added, turning the color from emerald green to yellow-greenish. The reaction mixture was left under stirring at 70 °C for another 2 h and then at room temperature overnight. The clear yellow-greenish solution, which under UV lamp irradiation showed a yellow-orange emission, was evaporated by vacuum to dryness. The obtained fine solid was dissolved in a few mL of dry THF and treated with an excess of diethyl ether (Et_2_O, 2×), obtaining a fine yellow precipitate. Yield 94% (124 mg). ^1^H NMR (D_2_O, 300 K, 9.4 T): δ phen-NH_2_ ligand 8.62 (1H, CH(9)), 8.35 (1H, CH(5)), 8.32 (1H, CH(7)), 8.20 (1H, CH(2)), 8.18 (1H, CH(6)), 7.72 (1H, CH(8)), 7.60 (1H, CH(3)), 3.30 (2H, CH(δ)), 2.93 (2H, CH_2_(α)), 1.83 (2H, CH_2_(γ)), 1.73 (2H, CH_2_(β)); δ phenylpyridine ligands 8.1 (2H, CH(6′)), 7.86 (2H, CH(3″)), 7.74 (2H, CH(5′)), 7.43 (2H, CH(3′)), 7.1 (2H, CH(4″)), 6.96 (2H, CH(5″)), 6.81 (2H, CH(4′)), 6.47 (2H, CH(6″)). The complete NMR characterization of this complex can be found in ref [^25^]. UV–vis (MeOH) MLCT absorptions: λ_abs_ = 377 nm (ε = 5102 M^–1^ cm^–1^) and 415 nm (ε = 2517 M^–1^ cm^–1^).

### 4.8 Synthesis of the Phen-COOH Ligand

Succinic anhydride (19.9 mg, 0.199 mmol) was dissolved in freshly distilled CH_2_Cl_2_ (1 mL) and treated with TEA (56 μL, 2.1 equiv). **Phen-NH**_**2**_ (50.0 mg, 0.199 mmol) was added to the solution. The mixture was refluxed under stirring for 6 h and then at room temperature overnight. The next day, the mixture presented a small amount of a light brown sticky solid at the bottom of the flask and a light yellow clear supernatant solution. Then, the supernatant was isolated and the sticky solid was washed with a few mL of CH_2_Cl_2_ (2×) warming at 40 °C. The crude product, present in the combined supernatants, was characterized by NMR in CH_2_Cl_2_/CDCl_3_. ^**1**^**H NMR** (CH_2_Cl_2_/CDCl_3_, 300 K, 9.4 T): δ 8.88 (1H, C*H*(9) dd, J = 4.5 Hz, 1.9 Hz), 8.7 (1H, C*H*(2) d, J = 4.7 Hz), 8.0 (1H, C*H*(7) dd, J = 8.2 Hz, 1.9 Hz), 7.8 (1H, C*H*(5) d J = 9.2 Hz), 7.6 (1H, C*H*(6)d J = 9.2 Hz), 7.3 (1H, C*H*(8) dd J = 8.2 Hz, 4.5 Hz), 7.2 (1H, C*H*(3) d J = 6.6 Hz), 7.45 (1H, CON*H*(5′)), 3.0 (2H, C*H*_2_(4′) m), 2.9 (2H, C*H*_2_(1′) t J = 19.2 Hz), 2.2 (4H, C*H*_2_(7′ and 8′) m), 1.5 (2H, C*H*_2_(2′) m), 1.4 (2H, C*H*_2_(3′) m). ^**13**^**C NMR** (CH_2_Cl_2_/CDCl_3_, 300 K, 9.4 T): δ 178.4 (*C*OO(9′)), 173.5 (*C*ONH(6′)), 149.8 (*C*H(9)), 149.5 (*C*H(2)), 148.4 (*C*_q_(1a)), 146.4 (*C*_q_(10a)), 145.9 (*C*_q_(4a)), 135.6 (*C*H(7)), 128.2 (*C*_q_(6a)), 127.5 (*C*_q_(4)), 125.9 (*C*H(6)), 122.8 (*C*H(3)), 122.6 (*C*H(8)), 122.2 (*C*H(5)), 38.4 (*C*H_2_(4′)), 32.9 (*C*H_2_(7′ and 8′)), 31.8 (*C*H_2_(1′)), 29.4 (*C*H_2_(3′)), 27.5 (*C*H_2_(2′)). Et_2_O was added to the combined supernatants, which caused the precipitation of a whitish solid. The mixture was evaporated to dryness, the residue was washed with CH_2_Cl_2_/Et_2_O, and the resulting whitish crude precipitate was isolated and vacuum-dried. Yield 49.6%. Elemental Analysis: Found = C, 62.52%; H, 6.62%; N, 10.74% (calcd for (C_20_H_21_N_3_O_3_)(C_4_O_4_H_6_)_0.45_(C_6_H_15_NCl)_0.6_: C, 62.70%; H, 6.77%; N, 10.36%).

### 4.9 Synthesis of the Ir-COOH Complex

To a suspension of **Phen-COOH** (37 mg, 0.105 mmol) in freshly distilled THF (20 mL) in a Schlenk tube, the precursor [Ir(ppy)_2_Cl]_2_ was added (48.1 mg, 0.045 mmol, 0.43 equiv) under stirring at room temperature. The mixture was heated at 70 °C under magnetic stirring and an inert atmosphere. Neither the Ir precursor nor the functionalized phenanthroline was soluble, even when the mixture was heated at reflux. Hence, after 30 min at 70 °C, the mixture was cooled down at room temperature and added with 6 mL of H_2_O Milli-Q. Immediately the heterogeneous mixture became more homogeneous, and after a few minutes at 70 °C the mixture turned clear and yellow. The mixture was left under stirring at 70 °C for ca. 5 h and then was left overnight at room temperature. The obtained clear yellow mixture, which under UV lamp irradiation showed a yellow-orange emission, was evaporated by vacuum to dryness, and the residue was redissolved in a few mL of dry THF and treated with an excess of Et_2_O (2x) to give a fine yellow precipitate. Yield 96.3% (80.2 mg, of which 3.1 mg are triethylammonium succinate). ^**1**^**H NMR** (MeOD, 300 K, 9.4 T): δ phen-COOH ligand 8.76 (1H, C*H*(9) dd, J = 8.2 Hz, 1.9 Hz), 8.52 (1H, C*H*(5) d, J = 9.2 Hz), 8.37 (1H, C*H*(7) dd, J = 5.3 Hz, 1.9 Hz), 8.32 (1H, C*H*(6) d, J = 9.2 Hz), 8.24 (1H, C*H*(2) d, J = 5.6 Hz), 7.90 (1H, C*H*(8) dd J = 8.2 Hz, 5.3 Hz), 7.79 (1H, C*H*(3) d, J = 5.6 Hz), 3.36 (2H, C*H*_*2*_(1″) t, J = 8.9 Hz), 3.29 (2H, C*H*_*2*_(4″) t, J = 6.8 Hz), 2.54 (2H, C*H*_*2*_(8″) t, J = 7.0 Hz), 2.43 (2H, C*H*_*2*_(7″) t, J = 7.0 Hz), 1.86 (2H, C*H*_*2*_(3″) m), 1.70 (2H, C*H*_*2*_(2″) m); δ phenylpyridine ligands 8.13 (1H, C*H*(2′) *ps*d, J = 8.2 Hz), 7.87 (1H, C*H*(9′) *ps*d J = 7.9 Hz), 7.81 (1H, C*H*(3′) *ps*t, J = 7.7 Hz), 7.45 (1H, C*H*(5′) *ps*d, J = 5.9 Hz), 7.10 (1H, C*H*(8′) *ps*t, J = 7.5 Hz), 6.95 (1H, C*H*(7′) *ps*t, J = 7.7 Hz), 6.91 (1H, C*H*(4′) *ps*t, J = 6.4 Hz), 6.41 (1H, C*H*(6′) dd, J = 7.4, 2.8 Hz). ^**13**^**C NMR** (MeOD, 300 K, 9.4 T): δ phen-COOH ligand 176.2 (*C*OO(9″)), 173.8 (*C*ONH(6″)), 153.1 (C_q_(4)), 150.9 (C_q_(6a)), 150.6 (CH(7)), 149.9 (*C*H(2)), 147.2 (*C*_q_(10a)), 138.2 (*C*H(9)), 131.7 ((*C*_q_(1a)), 130.6 (*C*_q_(4a)), 127.9 (*C*H(6)), 126.4 (*C*H(3)), 124.7 (*C*H(8)), 124.6 (*C*H(5)), 38.3 (*C*H_2_(4″)), 31.4 (*C*H_2_(1″)), 31.3 (*C*H_2_(7″)), 30.5 (*C*H_2_(8″)), 29.0 (*C*H_2_(2″)), 27.2 (*C*H_2_(3″)). δ phenylpyridine ligands 168.1 (C_q_(12′)), 150.2 (C_q_(11′)), 148.6 (*C*H(5′)), 144.3 (*C*_q_(10′)), 138.3 (*C*H(3′)), 131.7 (*C*H(6′)), 130.2 (*C*H(7′)), 124.6 (*C*H(9′)), 123.0 (*C*H(4′)), 122.4 (*C*H(8′)), 119.6 (*C*H(2′)). ESI^+^ MS, *m*/*z*: 890.3 [M+K–H]^+^, 874.5 [M+Na–H]^+^, 852.7 [M]^+^, 752.6 [M–C(O)CH_2_CH_2_COOH]^+^. UV–vis spectroscopy of **Ir-COOH** in MeOH: ε (λ_abs_ = 377 nm) = 5.1 × 10^3^ M^–1^cm^–1^. ε (λ_abs_ = 415 nm) = 2.5 × 10^3^ M^–1^cm^–1^.

### 4.10 Synthesis of the Ir-PNA Conjugate through Coupling Reaction between PNA1-Fmoc and Ir-COOH

The **PNA1-Fmoc** resin (40 mg, 0.3 mmol/g, 12 μmol) was swollen in CH_2_Cl_2_ for 45 min, then was treated with a 20% *v/v* solution of piperidine in NMP (1 mL, twice for 8 min). A solution of HATU (14 mg, 36 μmol, 3 equiv) in NMP was added to a solution of the **Ir-COOH** (36 mg, 36 μmol, 3 equiv) and DIPEA (21 μL, 120 μmol, 10 equiv) in NMP, and the resulting solution was shaken for 2 min. Then, this brown solution was transferred to the resin that was shaken for 4 h at room temperature. The resin was washed with NMP, and the simultaneous deprotection of nucleobases and the cleavage were performed by treating the resin with a mixture of TFA/*m*-cresol (9/1, *v/v*, 1 mL) for 1.5 h. The collected filtrate was concentrated by bubbling N_2_ onto the solution, and the residue was poured onto cold Et_2_O to precipitate the crude **Ir-PNA** as a yellow solid. The solid was centrifuged, washed with Et_2_O, dried under a vacuum, and purified by RP-HPLC. HR-ESI^+^ MS, *m*/*z*: found (calculated) for C_85_H_91_N_29_O_14_Ir: 1932.6938 (1932.6906) [M]^+^, 1000.8215 (1000.8231) [M+3Na-2H]^2+^, 997.8154 (997.8191) [M+Na+K–H]^2+^, 989.8307 (989.8322) [M+2Na–H]^2+^, 978.8405 (978.8412) [M + Na]^2+^, 966.8480 (966.8487) [M + H]^2+^, 665.5449 (665.5485) [M+Na+K]^3+^, 660.2238 (660.2239) [M+2Na]^3+^, 658.2186 (658.2212) [M+K+H]^3+^, 652.8962 (652.8966) [M+Na+H]^3+^, 644.9016 (644.9015) [M+2H]^3+^. Analytical RP-HPLC: *t*_R_ = 29.6 min.

### 4.11 Synthesis of the Ir-PNA Conjugate through Coupling Reaction between PNA2 and Ir-NH_2_

After the swelling in CH_2_Cl_2_ for 45 min, **PNA2** resin (40 mg, 0.3 mmol/g, 12 μmol) was shaken with a solution of HATU (14 mg, 36 μmol, 3 equiv) and DIPEA (21 μL, 120 μmol, 10 equiv) in NMP for 8 min. Then, the brown solution of the **Ir-NH**_**2**_ (28 mg, 36 μmol, 3 equiv) was transferred to the resin containing the solution of HATU and DIPEA and shaken for 4 h at room temperature. The resin was washed with NMP, and after the cleavage, the crude **Ir-PNA** was obtained as a yellow solid, and its RP-HPLC analysis showed a very similar trend observed for the conjugation with the **Ir-COOH**. Analytical RP-HPLC: *t*_R_ = 29.6 min.

### 4.12 Stability Test on Ir-NH_2_ under Cleavage Conditions

A tiny amount of the **Ir-NH**_**2**_ complex was dissolved in 3 mL of a TFA/*m*-cresol 9:1 mixture, and the solution was monitored by UV–vis spectroscopy, recording subsequent spectra over a period of 1.5 h, mimicking the contact time and the conditions used during the cleavage of the PNA from the resin. UV–vis spectra are reported in Figure S12.

### 4.13 Photochemical Stability of Ir-COOH and Ir-PNA

The **Ir-COOH** complex and the **Ir-PNA** conjugate, both dissolved in methanol, were diluted to 2.5 mL by using a CH_2_Cl_2_–methanol mixture (9:1) in a quartz cuvette. In this way, the same concentration used during the photoreaction of the two photosensitizers with DHN was achieved. The solutions were irradiated with a monochromatic LED light (400 nm, 15.2 mW/cm^2^) for increasing times ranging from 1 to 25 min. Potential variations were monitored by UV–vis spectroscopy (Figures S22 and S23).

### 4.14 Photoreaction of Ir-COOH and Ir-PNA with DHN

In a quartz cuvette, 15 μL of a 1.68 × 10^–3^ M methanol solution of the **Ir-PNA** adduct was diluted to 2.25 mL with a CH_2_Cl_2_-methanol 9:1 mixture. 250 μL of a 1.44 × 10^–3^ M solution of DHN was added in the same solvent mixture, to obtain a 1:10 PS-DHN molar ratio. Subsequently, the solution was saturated with O_2_ by bubbling it for 5 min and exposed to monochromatic LED light (400 nm, 15.2 mW/cm^2^) for durations ranging from 30 s to 25 min. Thus, the variations in DHN and Juglone absorption bands were monitored using UV–vis spectroscopy (Figure 3 of the main text); in particular, the decreasing absorbance of the DHN band at 316 nm was used for the determination of the initial rate constant (*k*_obs_) of the oxidation of DHN to Juglone. The same procedure was carried out for the **Ir-COOH** complex and for the **Ir(ppy)**_**3**_, whose ^1^O_2_ quantum yield (Φ_Δ_) in CH_2_Cl_2_-methanol 9:1, obtained from the literature,^34^ was used as a standard to calculate the Φ_Δ_ values for the **Ir-PNA** and the **Ir-COOH**. While the equation rate of the overall reaction of juglone production can be described by eq 1, by applying the steady-state approximation to the reactive ^1^O_2_ species, the DHN consumption reaction in the initial stages (*v*) follows pseudo-first-order kinetics, as described by eq 2.
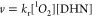
1
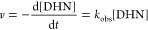
2

The ^1^O_2_ production ability of the investigated species can thus be indirectly monitored by evaluating the corresponding *k*_obs_ rate constant, which can be estimated by plotting, as a function of the irradiation time, the characteristic DHN absorbance value (*A*), registered at 316 nm, according to the following integrated eq (eq 3):
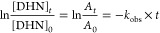
3

The fitted absorbance values (with *A_t_* and *A*_0_ referring to the *A* values measured at times t and 0, respectively) were thus obtained from regularly acquired UV–vis spectra of the reaction mixture over a fixed irradiation time, as depicted in Figure 3. Panel (d) of Figure 3 shows a comparison of the linear decrease of ln(A_t_/A_0_) vs time acquired for the investigated systems. The quantum yield for singlet oxygen ^1^O_2_ formation (Φ_Δ_) can then be determined by using eq 4:
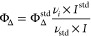
4where *v*_*i*_ is the initial rate, *I* indicates the photons absorbed by the sensitizer, and the apex std labels the values for the adopted standard, which in our case corresponds to the Ir(ppy)_3_ complex, whose generation of ^1^O_2_ in DCM/methanol 9:1 is known and fixed to Φ_Δ_ = 0.50.^34^ The values of ν_i_ were obtained as the product of *k*_obs_ and [DHN]_0_ (with [DHN]_0_ in the 1.65–1.70 × 10^–4^ M range), while the value of *I* was calculated as *I* = *I*_source_ (400 nm) × (1–10^*A*(400 nm)^) where *I*_source_ (400 nm) is the intensity of the 400 nm LED emitted light incident on the sample, while *A*(400 nm) is the corresponding absorbance of the sensitizer at such wavelength.

### 4.15 Cell Culture and Treatment

HeLa cells were cultured in DMEM culture medium (EuroClone, ECB7501) supplemented with 1 mM l-glutamine (EuroClone, ECB3004D), penicillin-streptomycin (SERVA 31749.04, 35500.01), and 10% supplemented with 10% fetal bovine serum (GIBCO, A4766801) at 37 °C in 5% CO_2._ For fluorescence microscopy analysis, HeLa cells were seeded at 200,000 cells/well in 6-well plates (Euroclone, ET3006) and treated with 25 μM **Ir-COOH** or **Ir-PNA**, and methanol as vehicle control for 24 h. For cell viability assay, HeLa cells were seeded at 25,000 cells/well in 48-well plates (Euroclone, ET3048) and treated for 24 h with different doses of the **Ir-COOH** and the **Ir-PNA** (1–5–10–25–50 μM). Untreated cells and cells treated with methanol (vehicle) were used as negative controls.

### 4.16 Viability Tests in the Dark and in Light

MTT (3-(4,5-dimethylthiazolyl-2)-2,5-diphenyltetrazolium bromide) (M5655, Sigma-Aldrich) assay was performed to determine the cell viability and the potential cytotoxicity of the **Ir-COOH** and the **Ir-PNA** both in the dark and under irradiation using a nail UV lamp. After 24 h of treatment, cells were exposed to light for 30 min, and after 6 h of incubation at 37 °C, the medium was replaced with 300 μL of MTT solution. Following incubation for 30 min at 37 °C, MTT was removed and 500 μL of 2-propanol was added to each well to dissolve formazan crystals. The same experiments were performed in the dark as the control. Formazan formation was measured spectrophotometrically at 570 nm using the microplate reader (PerkinElmer, EnSpire, Waltham, MA, USA). Light EC_50_ values were obtained by analyzing the fitting curves by OriginPro2021 (Copyright © 1991–2021 OriginLab Corporation) software using the vertical cursor instrument for reading the data at 50% of cell viability. **Statistical analysis:** Curve-fitting was performed with OriginPro2021 software, and statistical data analysis was performed using GraphPad Prism 9 (GraphPad Software Inc., San Diego, CA). Data were normalized to untreated control. Results were plotted using nonlinear regression with three parameters. One-way ANOVA corrected for multiple comparisons by Sidak′s post hoc analysis was performed, ***p < 0.001; *n* = 4.

## Supporting Information Available

Additional experimental details (docx), 1D and 2D NMR spectra (MestrecNova metafiles), MS spectra (PDF), UV–vis spectra (excel or Origin figures), emission spectra (Origin figures), lifetime decays (WMF), DLS (Origin figures), and lamp profiles (excel figures) (PDF)

## Notes

Mass spectrometry analyses were performed at the Mass Spectrometry facility of the Unitech COSPECT at the University of Milan (Italy). The use of instrumentation purchased through the Regione Lombardia-Fondazione Cariplo joint SmartMatLab Project is gratefully acknowledged.
