# Supplementary material for: Luminescent Iridium-Peptide Nucleic Acid Bioconjugate as Photosensitizer for Singlet Oxygen Production toward a Potential Dual Therapeutic Agent
Source: Inorg Chem. 2025 Mar 27;64(14):6898–911. doi: 10.1021/acs.inorgchem.4c05359 (PMC12001249; doi:10.1021/acs.inorgchem.4c05359)
Supplement: Supplementary file 1 — ic4c05359_si_001.pdf [file ic4c05359_si_001.pdf]

SUPPORTING INFORMATION

Luminescent iridium-peptide nucleic acid bioconjugate as photosensitizer for singlet oxygen production towards a potential dual therapeutic agent

Rosa Maria Dell'Acqua,[a] Veronica Schifano,[a] Maria Vittoria Dozzi,[a] Laura D'Alfonso,[b] Monica Panigati,[a,d] Paola Rusmini,[c] Margherita Piccolella,[c] Angelo Poletti,[c] Silvia Cauteruccio,[a,*] Daniela Maggioni[a,d,*]

[a] *Dipartimento di Chimica, Università degli Studi di Milano, Via Golgi 19, 20133 Milano, Italy.*

[b] *Dipartimento di Fisica "G. Occhialini", Università degli Studi di Milano-Bicocca, piazza della Scienza 3, 20126 Milano, Italy.*

[c] *Dipartimento di Scienze Farmacologiche e Biomolecolari "Rodolfo Paoletti", Dipartimento di Eccellenza 2018-2027, Università degli Studi di Milano, Via Balzaretti 9, 20133 Milano, Italy.*

[d] *Consorzio INSTM, Via G. Giusti 9, 50121 Firenze, Italy.*

daniela.maggioni@unimi.it, silvia.cauteruccio@unimi.it

TABLE OF CONTENT

I. NMR and/or mass spectra of **Phen-COOH** and **Ir-COOH** S2

II. Stability test on **Ir-NH$_2$** under cleavage conditions S13

III. ESI$^+$ MS spectrum and RP-HPLC trace of **Ir-PNA** S14

IV. Photophysical behaviour of **Ir-COOH** in different solvents S16

V. Dynamic Light Scattering measurement on **Ir-COOH** in MeOH S20

VI. Photochemical stability of **Ir-COOH** and **Ir-PNA** S21

VII. UV-LED and UV-lamp emissions S22

I. NMR and/or mass spectra of Phen-COOH and Ir-COOH

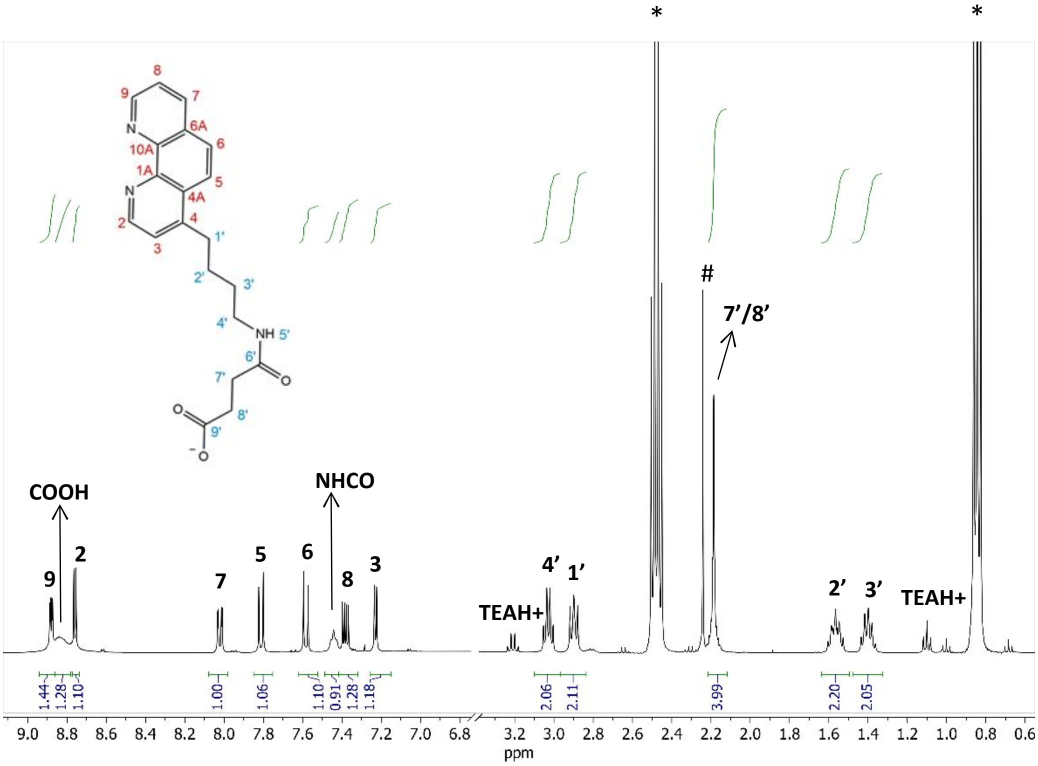

Figure S1. ^{1}H NMR spectrum of crude **Phen-COOH** in $CH_2Cl_2/CDCl_3$ (9.4 T, 300 K). Asterisks mark TEA signals, while # marks succinate residue.

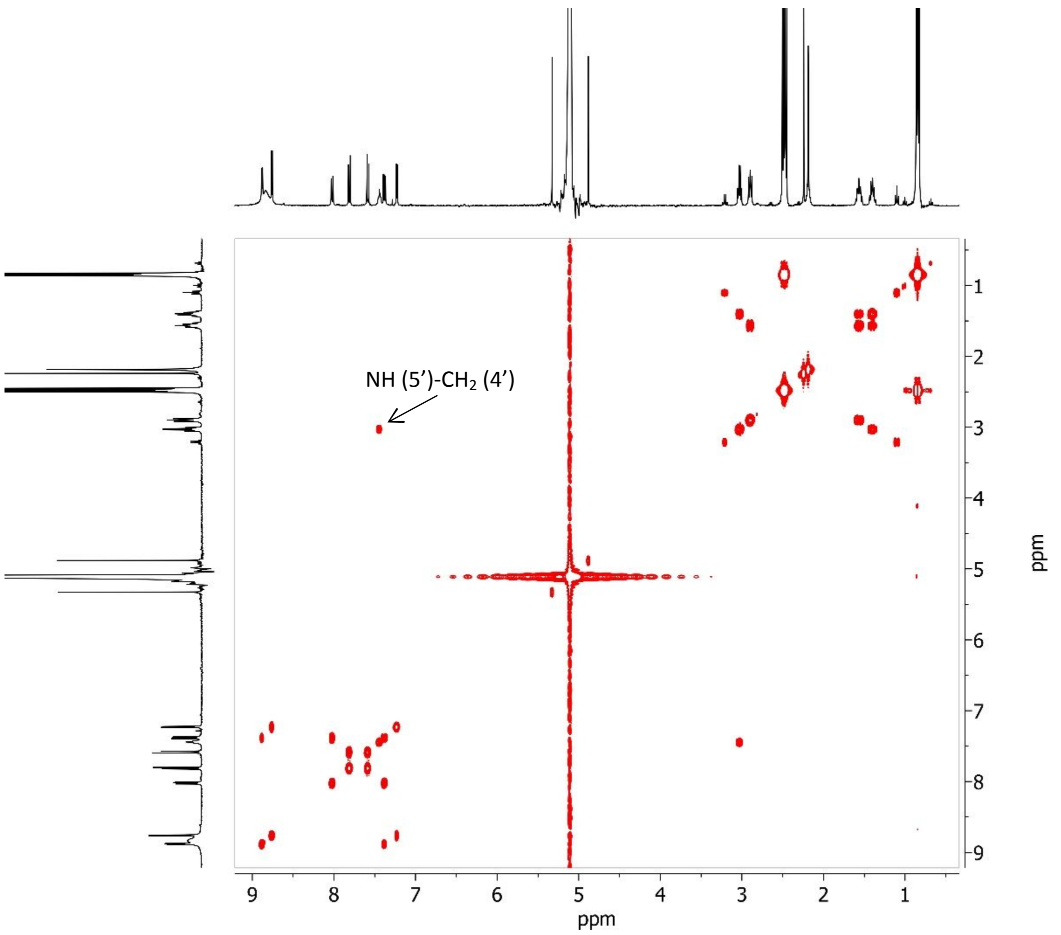

Figure S2. ^1H-^1H COSY NMR experiment on a sample of **Phen-COOH** in $CH_2Cl_2/CDCl_3$ (9.4 T, 300 K).

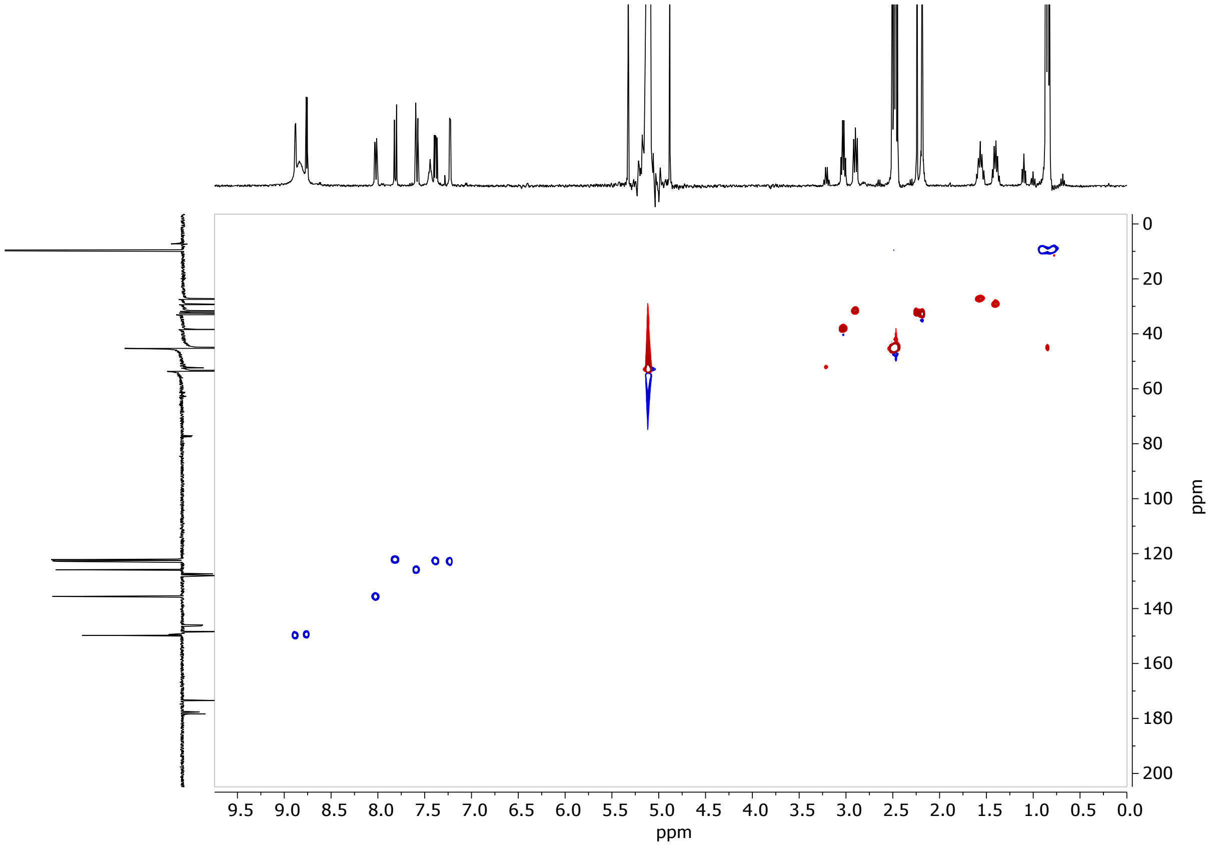

Figure S3. ^{1}H-^{13}C HSQC NMR experiment on a sample of **Phen-COOH** in $CH_2Cl_2/CDCl_3$ (9.4 T, 300 K).

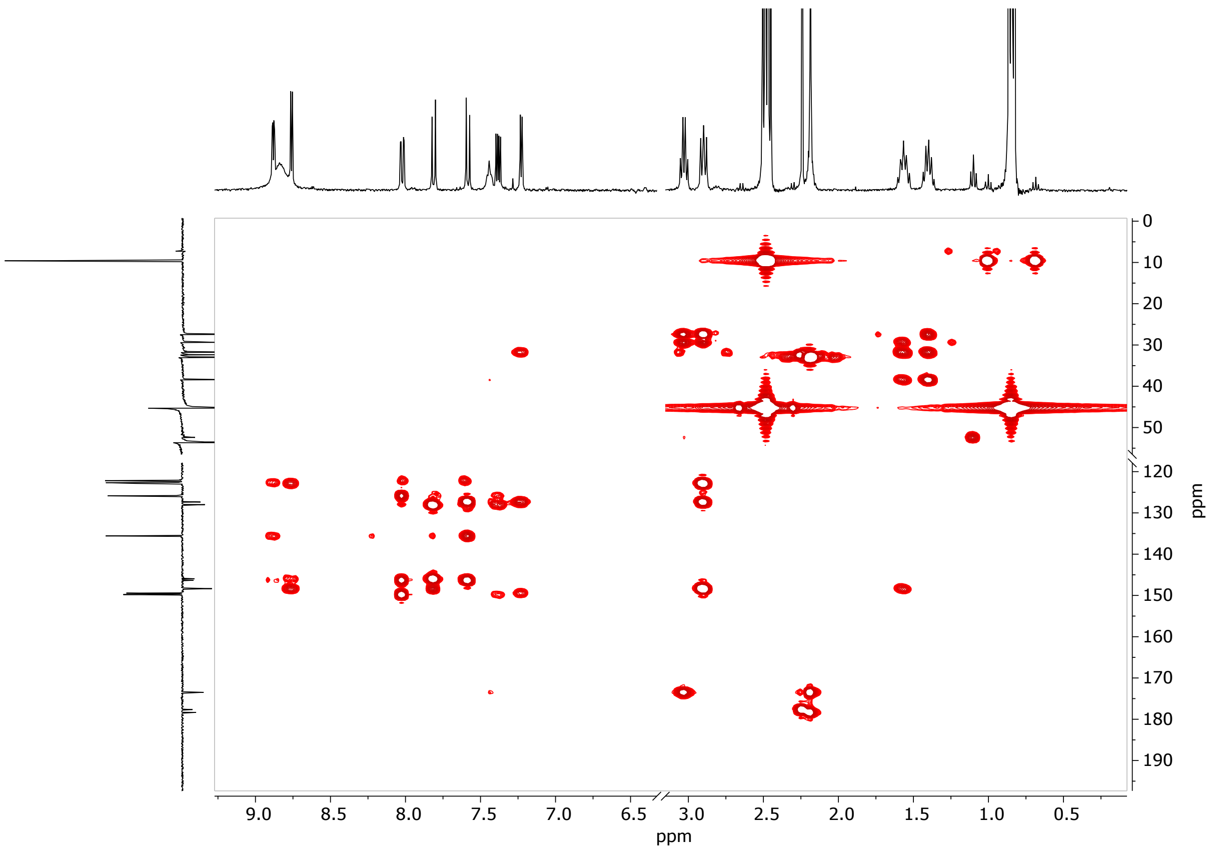

Figure S4. ^{1}H-^{13}C HMBC NMR experiment on a sample of **Phen-COOH** in $CH_2Cl_2/CDCl_3$ (9.4 T, 300 K).

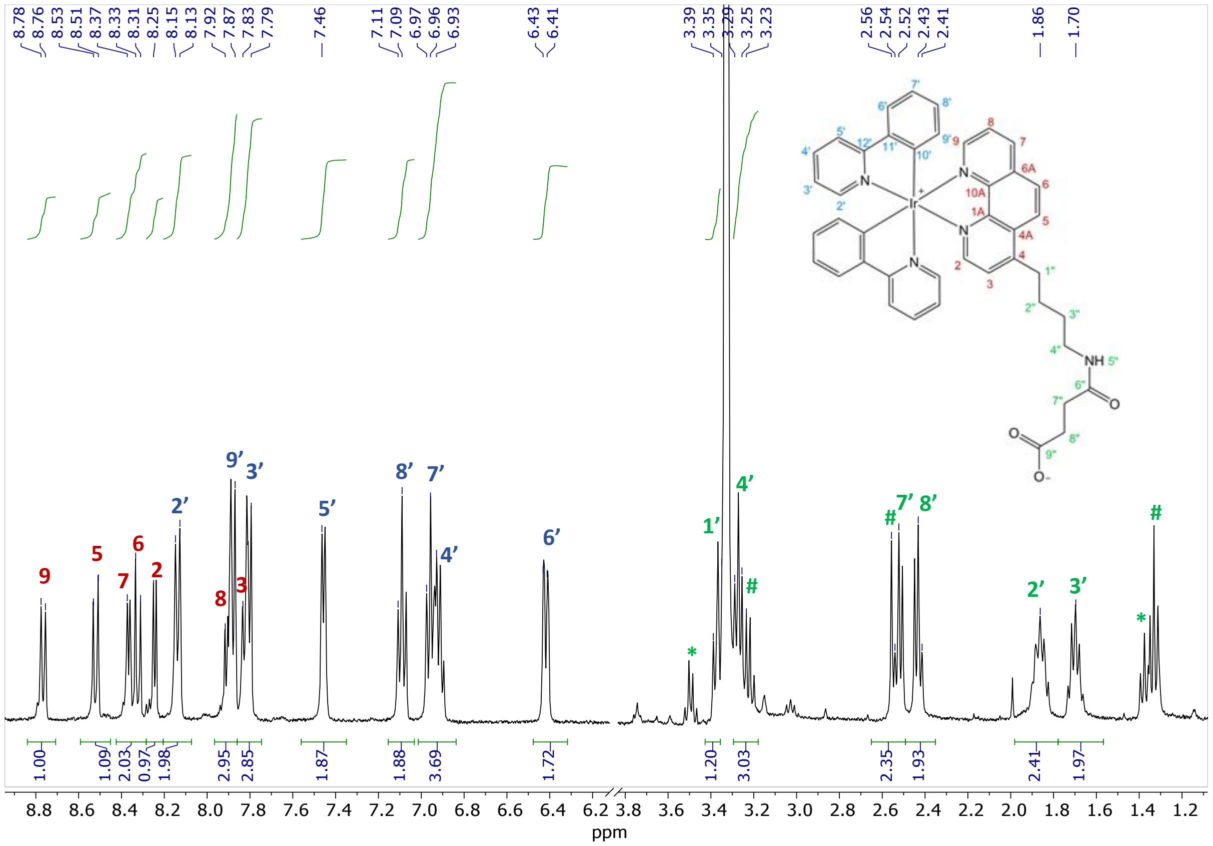

Figure S5. ^1H NMR spectrum of **Ir-COOH** in methanol-d_4 (9.4 T, 300 K, # = triethylammonium succinate; * = triethylammonium chloride).

The development of a NOE cross peak between CH_2 (1′) and CH_2 (2′) allowed the identification of the aromatic signals CH(5) and CH(3) of phenanthroline ligand. Consequently, it was easy to attribute signals of CH_2 (3′) and CH_2 (4′). NOE as well as the scalar correlations of ^1H COSY experiment developing with CH(5) enabled the identification of CH(6), and the same was achieved by analysing the cross peaks involving CH(3) that enabled the identification of CH(2). The most downfield shifted signal is attributed to CH(9), hence both scalar and dipolar correlations can be exploited to attribute signals of CH(8) and CH(7).

As to the phenyl pyridine ligand, the starting point was the attribution to the signal at 8.14 ppm to the CH(2′). Hence CH(3′), CH(4′) and CH(5′) were easily attributed by following the scalar and dipolar patterns. ^1H-^1H NOESY showed an extra dipolar correlation developed by CH(5′), which was assigned to the spatial interaction with CH(6′). Consequently, all the others CH of the phenyl ring have been easily identified.

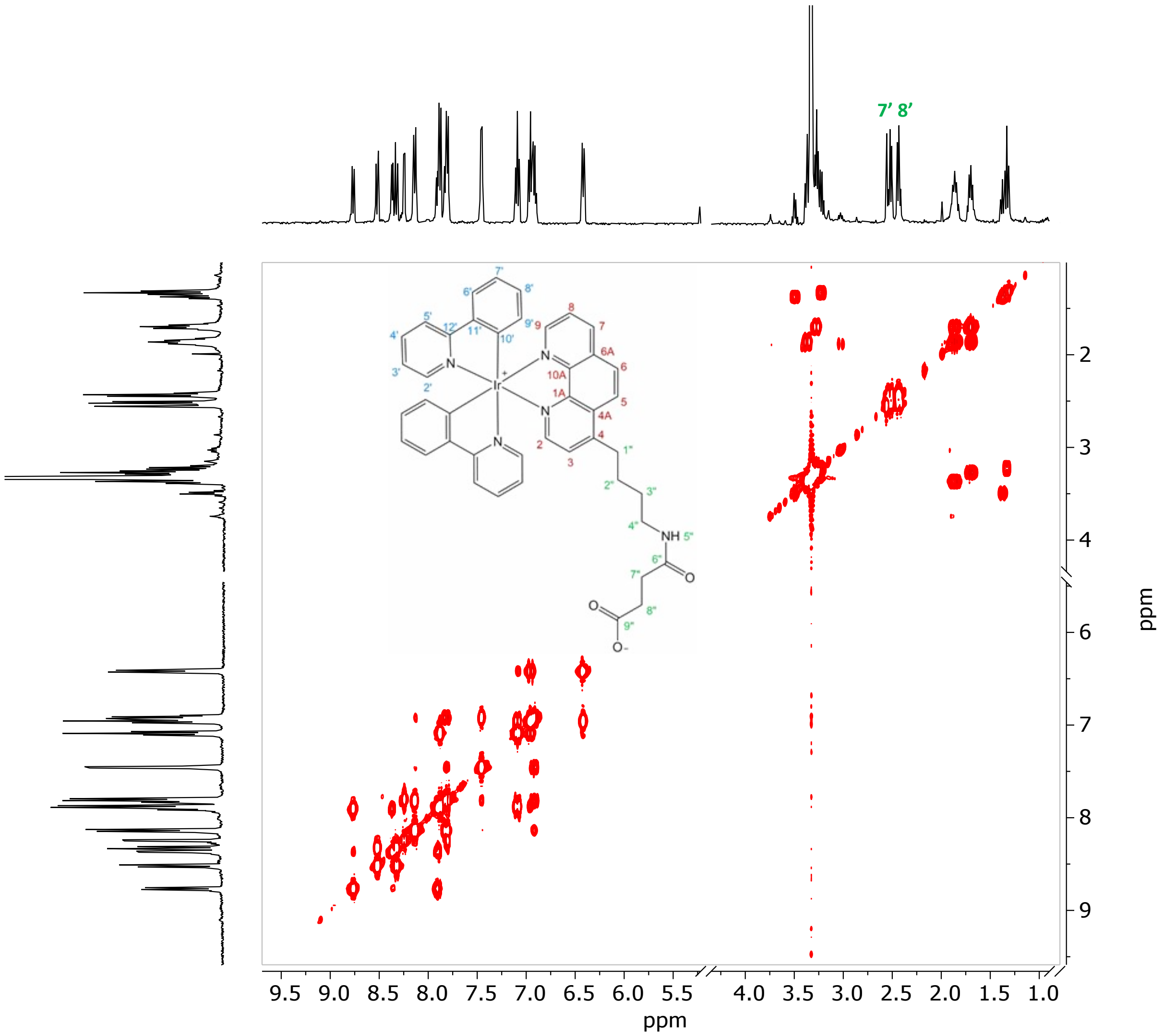

Figure S6. ^{1}H-^{1}H COSY NMR experiment on a sample of **Ir-COOH** in methanol-d_4 (9.4 T, 300 K).

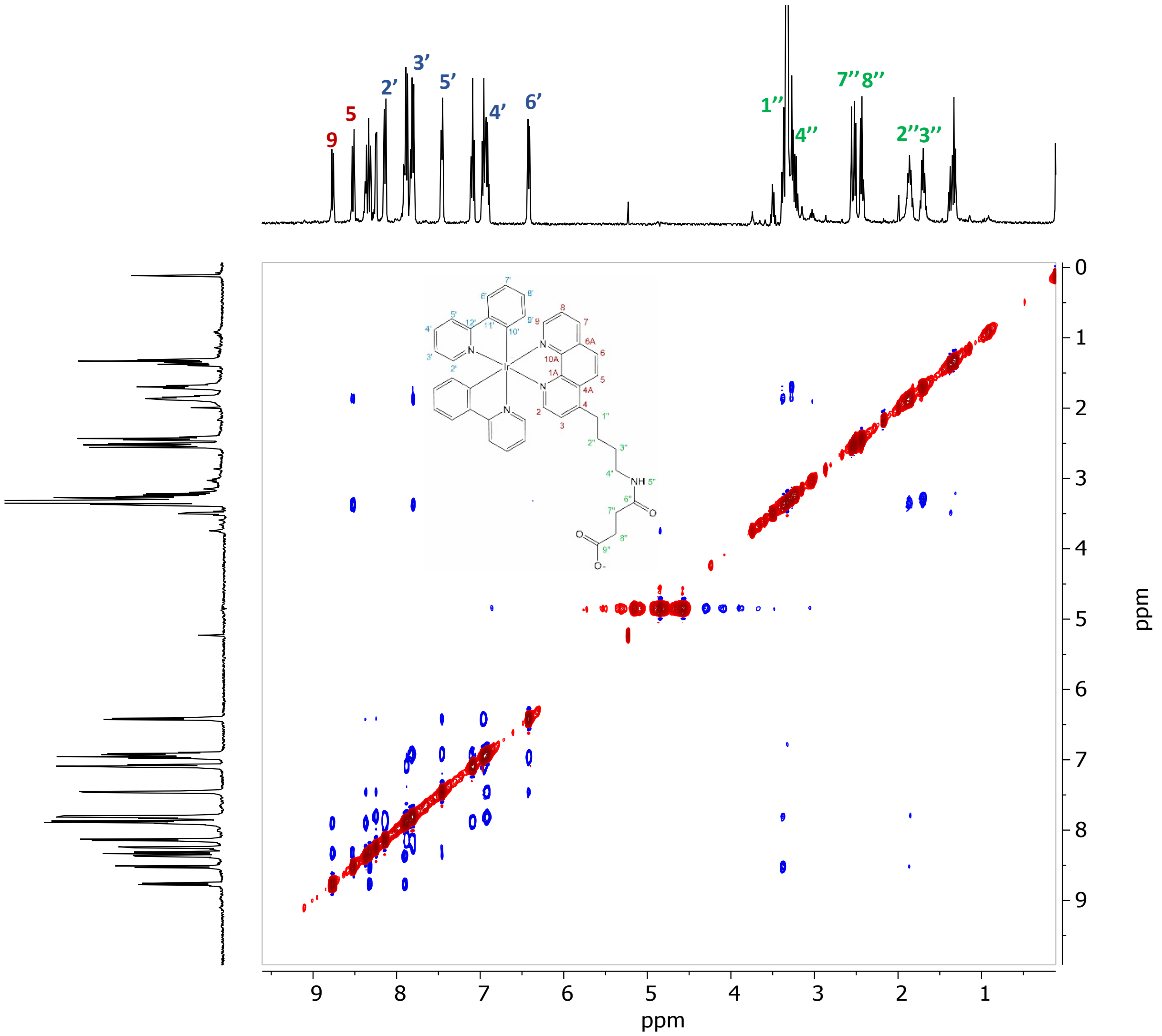

Figure S7. ^{1}H-^{1}H NOESY NMR experiment on a sample of **Ir-COOH** in methanol-d_4 (9.4 T, 300 K).

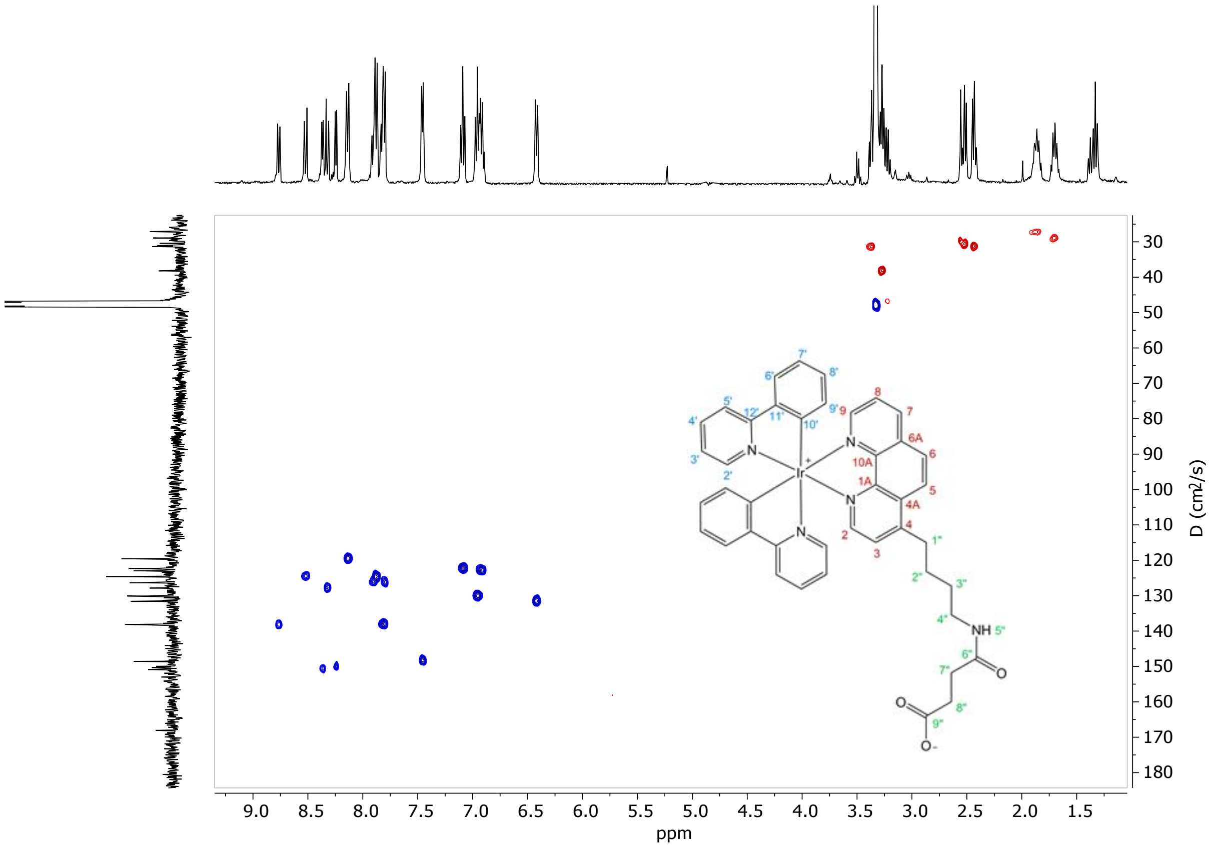

Figure S8. ^{1}H-^{13}C HSQC NMR experiment on a sample of **Ir-COOH** in methanol-d_4 (9.4 T, 300 K).

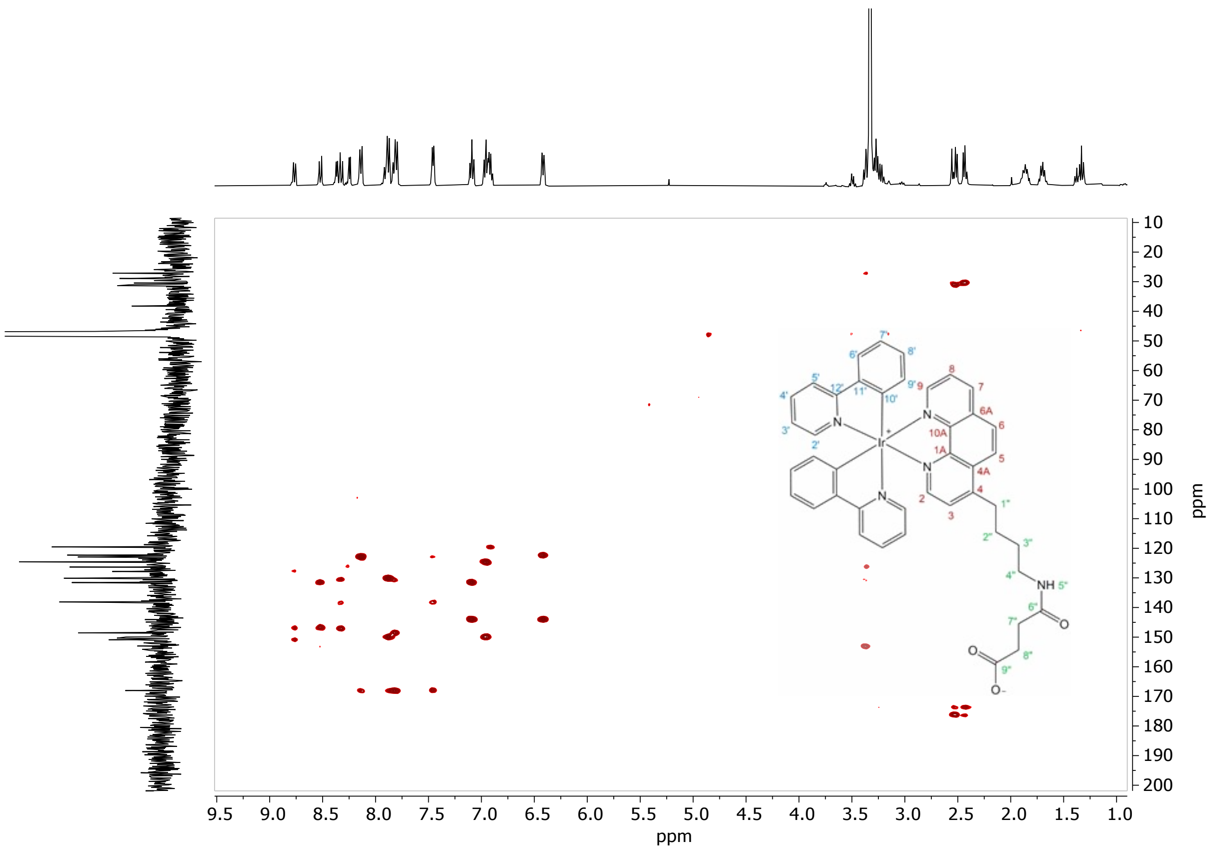

Figure S9. ^{1}H-^{13}C HMBC NMR experiment on a sample of **Ir-COOH** in methanol-d_4 (9.4 T, 300 K).

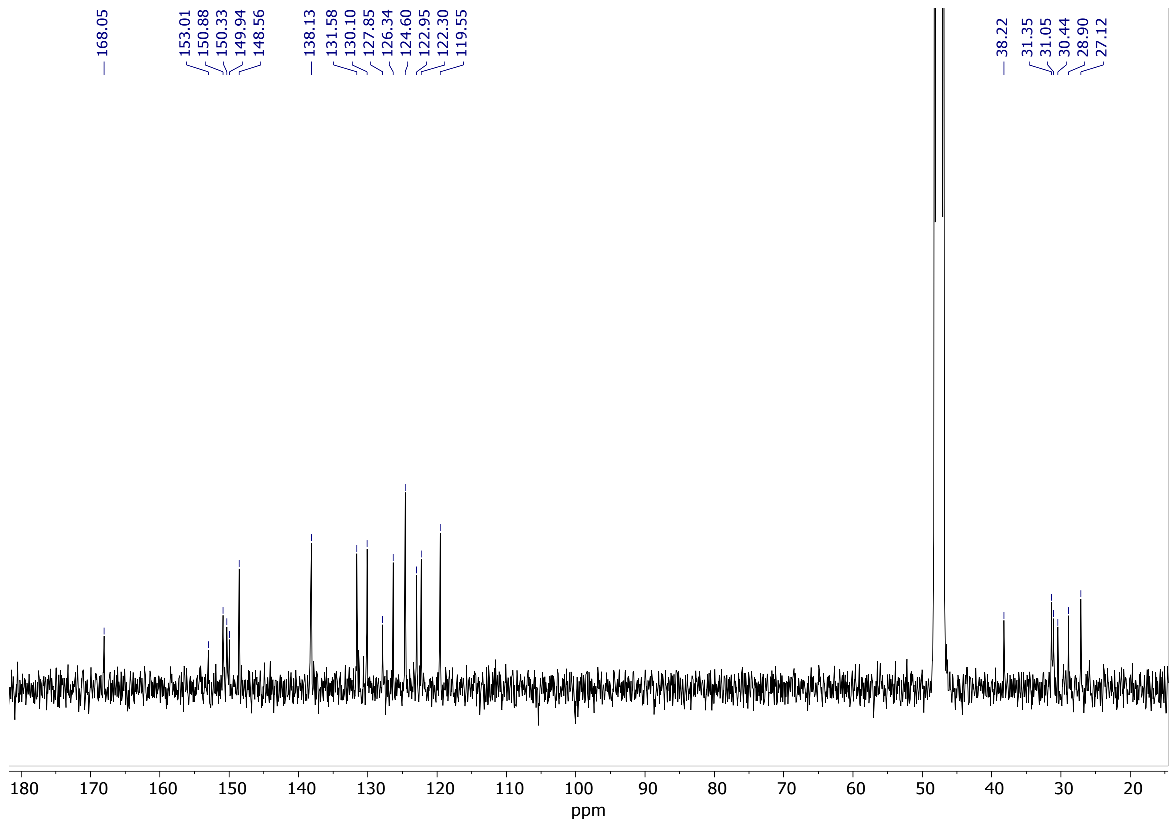

Figure S10. $^{13}C\{^{1}H\text{-}\}$ NMR experiment on a sample of **Ir-COOH** in methanol-d_4 (9.4 T, 300 K).

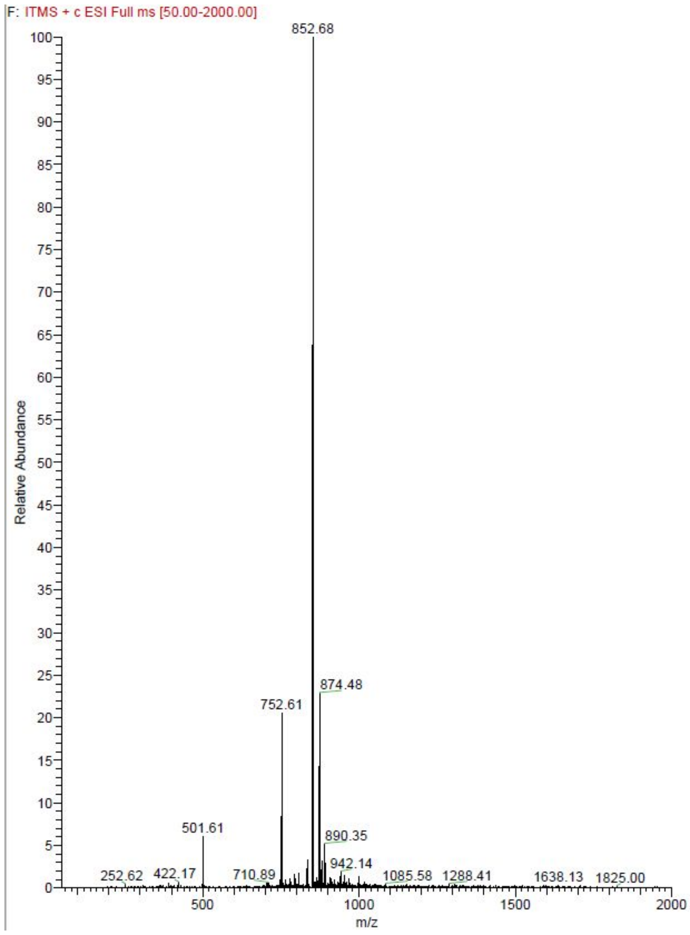

Figure S11. ESI^{+} MS spectrum of the **Ir-COOH**

II. Stability test on Ir-NH_2 under cleavage conditions

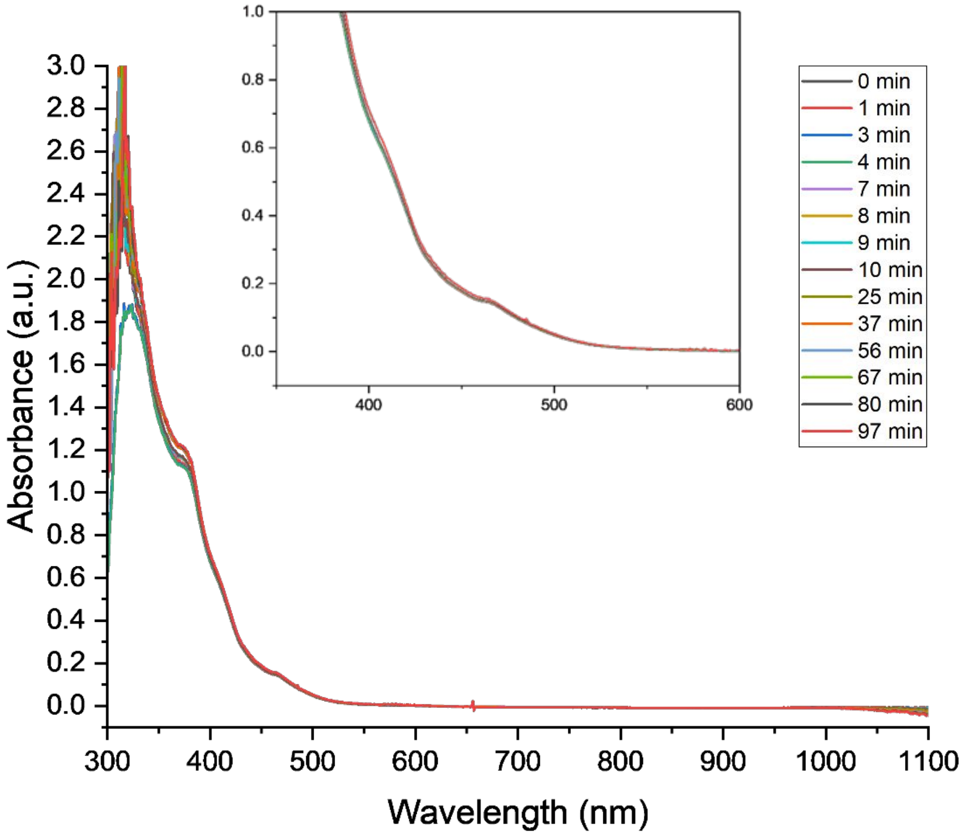

Figure S12. UV-vis spectra of complex **Ir-NH$_2$** in a mixture of TFA/*m*-cresol 9:1 at room temperature over 1.5 h.

III. ESI$^+$ MS spectrum and RP-HPLC trace of Ir-PNA

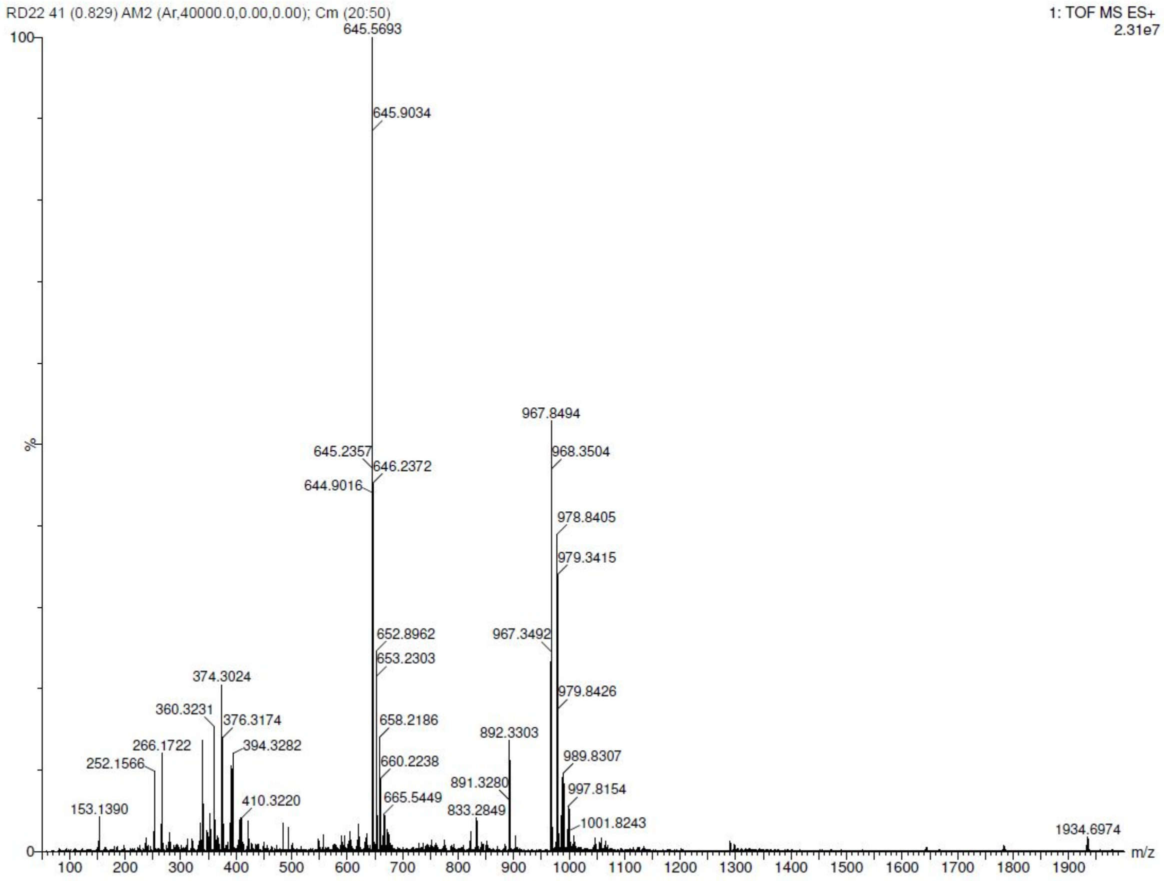

Figure S13. HR ESI$^+$ MS spectrum of the **Ir-PNA**.

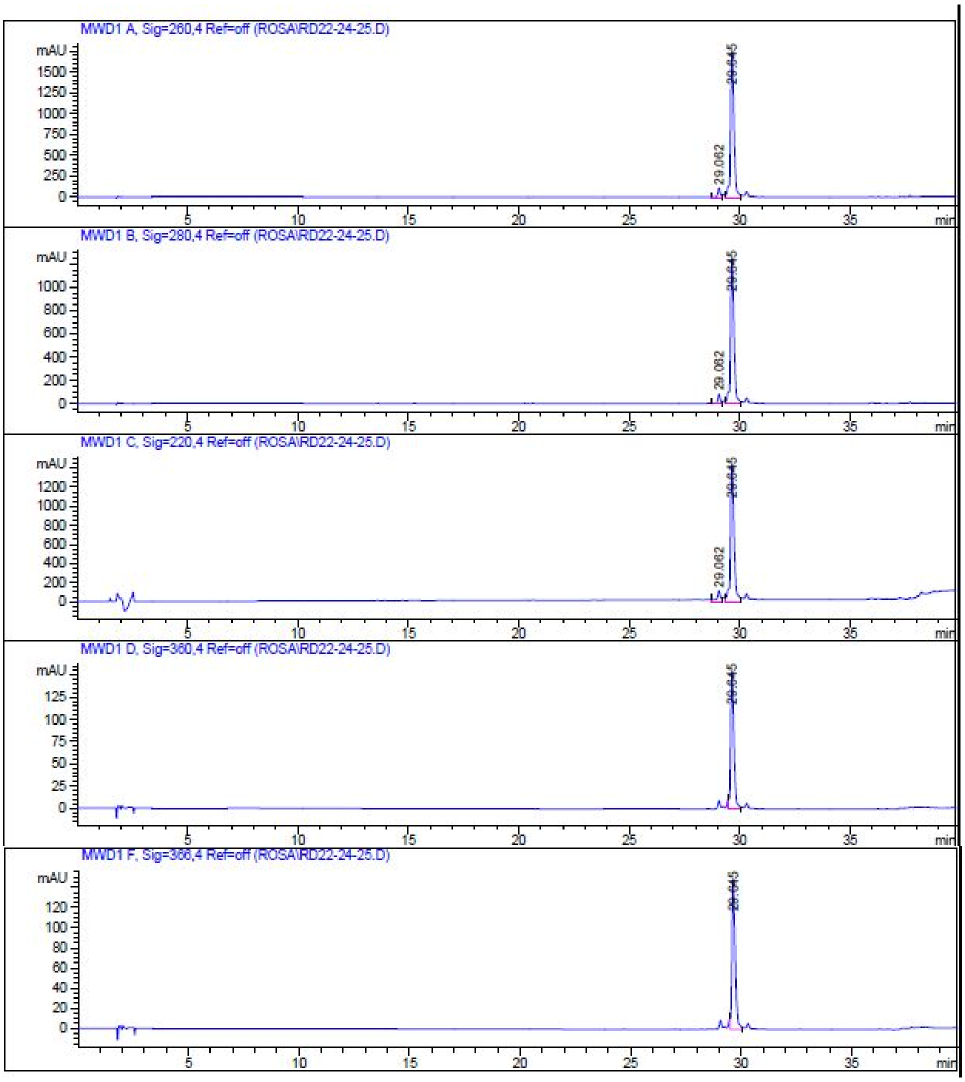

Figure S14. RP-HPLC trace of the **Ir-PNA** after purification.

IV. Photophysical behaviour of Ir-COOH in different solvents

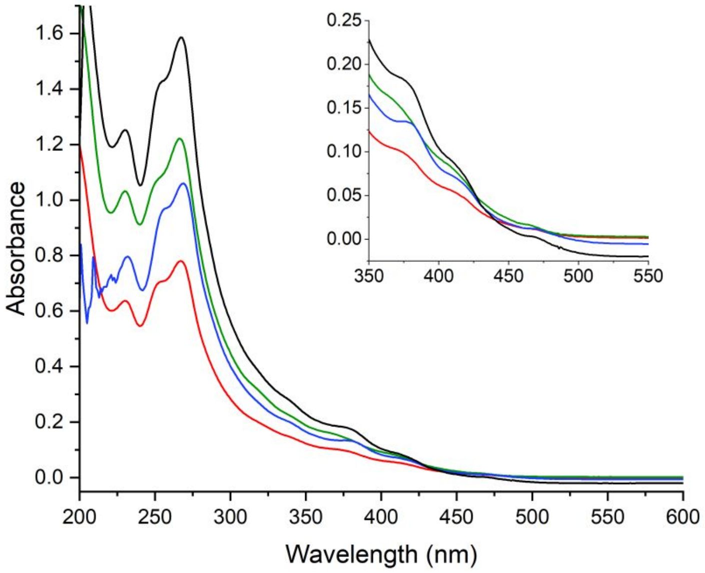

Figure S15. Absorption spectra of the **Ir-COOH** complex in methylene chloride (blue trace), acetonitrile (red trace), methanol (black trace) and water (green trace) at room temperature in aerated conditions.

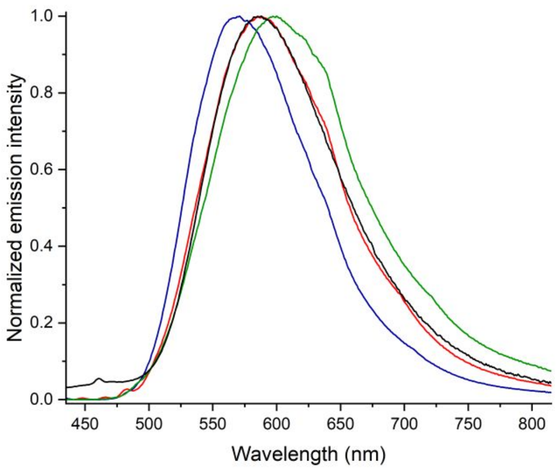

Figure S16. Photoluminescence spectra of the **Ir-COOH** complex in methylene chloride (blue trace), acetonitrile (red trace), methanol (black trace) and water (green trace) at room temperature in aerated conditions.

(a)

(b)

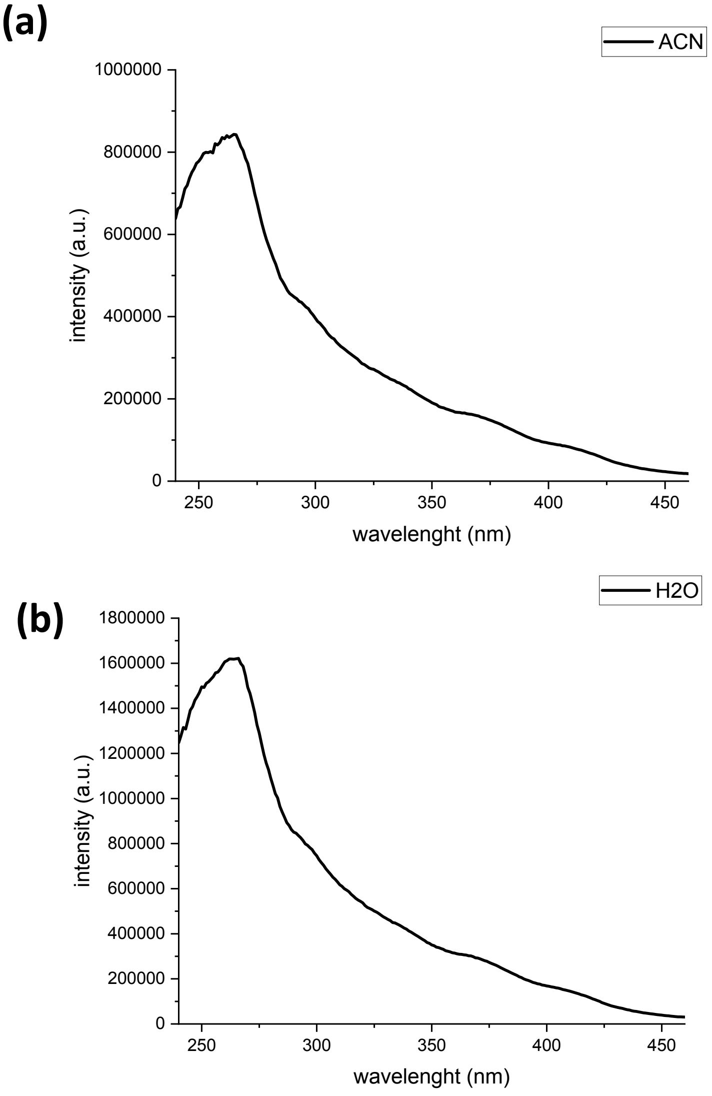

Figure S17. Excitation spectra of **Ir-COOH** dissolved in ACN (a) and H_2O (b).

Table S1. Photoluminescence data at room temperature for compound **Ir-COOH** in aerated mixtures of acetonitrile and water (1.1 x 10^{-5} M, λ_{ex} = 410 nm) and hydrodynamic diameter measured by DLS.

H_2O/CH_3CN volume ratio	λ_{em} (nm)	Φ	τ (ns)	k_r (s^{-1})	k_{nr} (s^{-1})	DLS (nm)
0:10	585	0.027	56.9	4.7×10^5	1.7×10^7	--
1:9	591	0.033	71.5	4.6×10^5	1.3×10^7	--
3:7	591	0.050	58 (4.6%)<br>103 (95.4%)	4.8×10^5	9.2×10^6	1.7 ± 0.7<br>255 ± 160
5:5	592	0.065	31 (1.3%)<br>138 (98.7%)	4.7×10^5	6.8×10^6	1.5 ± 0.4<br>295 ± 115
7:3	595	0.093	38(1%)<br>199 (99%)	4.7×10^5	4.5×10^6	105 ± 83
9:1	598	0.084	26 (0.7%)<br>185 (99.3%)	4.5×10^5	4.9×10^6	712 ± 326
10:0	600	0.051	31.8 (3%)<br>113.4 (97%)	4.5×10^5	8.4×10^6	1280 ± 476

[b] k_r and k_{nr} indicate the radiative and non-radiative decay constants of the excited states, respectively and are computed on the most significant lifetime component.

Samples were prepared by starting from a mother solution containing 0.89 mg of **Ir-COOH** in 1.00 mL of ACN. Apart, 10 mL of the five mixtures ACN/H_2O were prepared. Then, the final 1.1 x 10^{-5} M solutions were prepared directly in 5 quartz cuvettes adding 25 µL **Ir-COOH** mother solution in 2.25 mL of each of the ACN/H_2O mixtures.

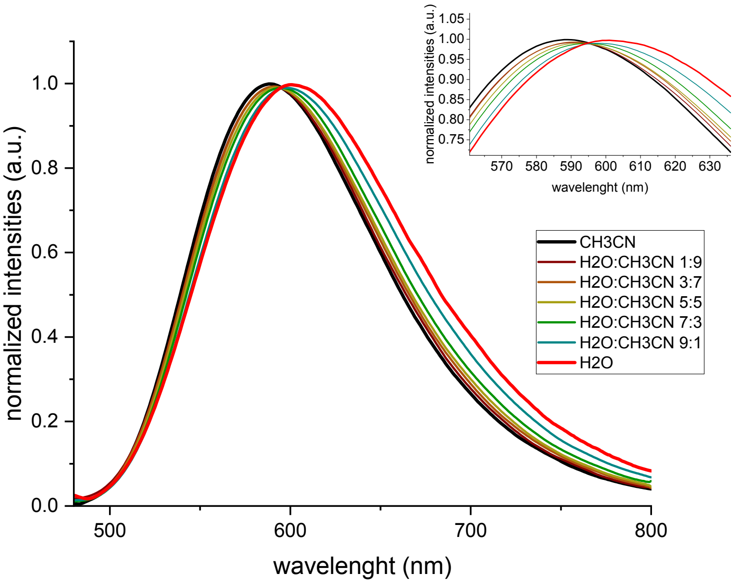

Figure S18. Photoluminescence spectra of the **Ir-COOH** complex in CH_3CN/H_2O mixtures.

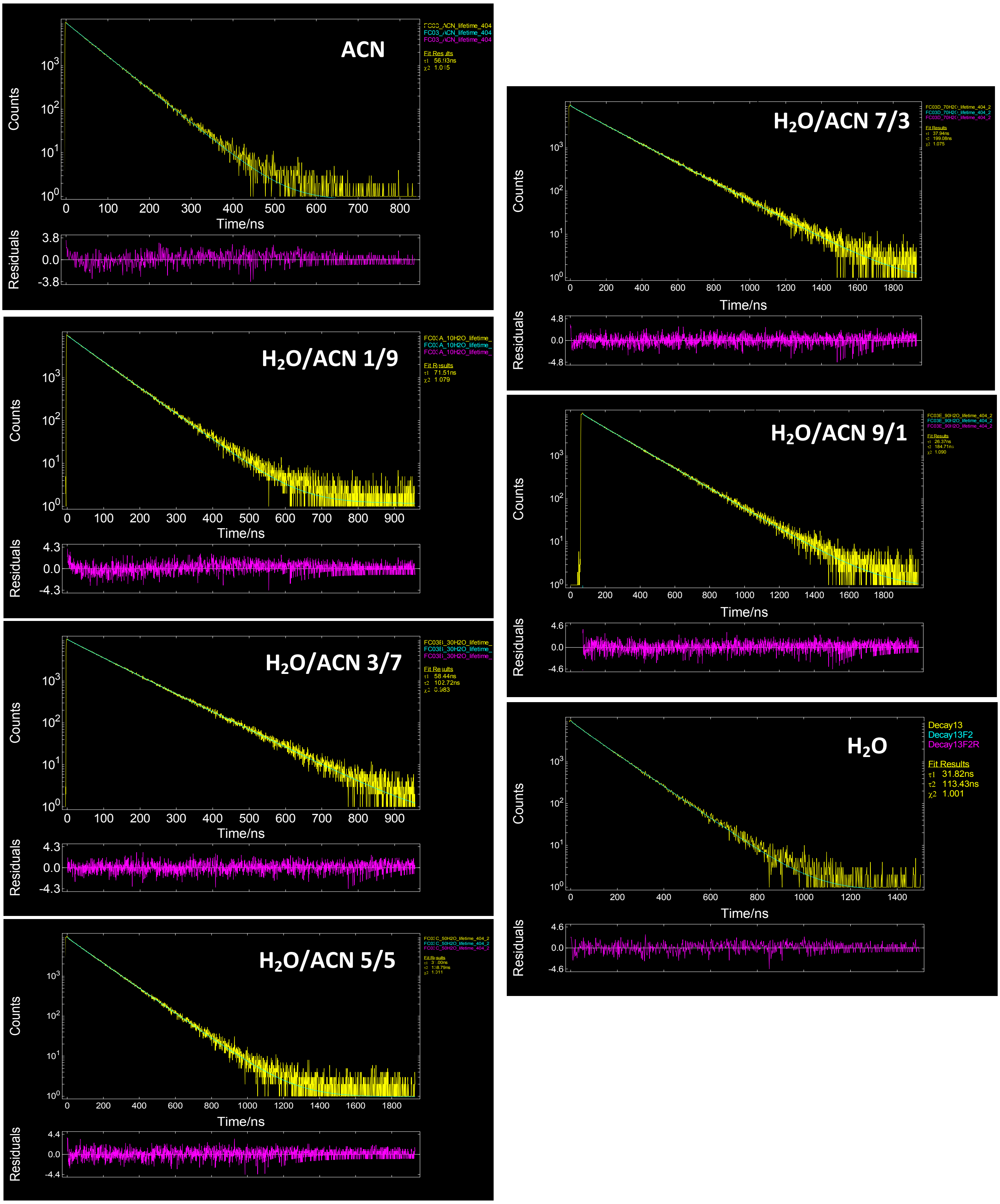

Figure S19. Fitted lifetime decays of the H_2O/ACN solutions of **Ir-COOH**.

V. Dynamic Light Scattering measurement on Ir-COOH in MeOH

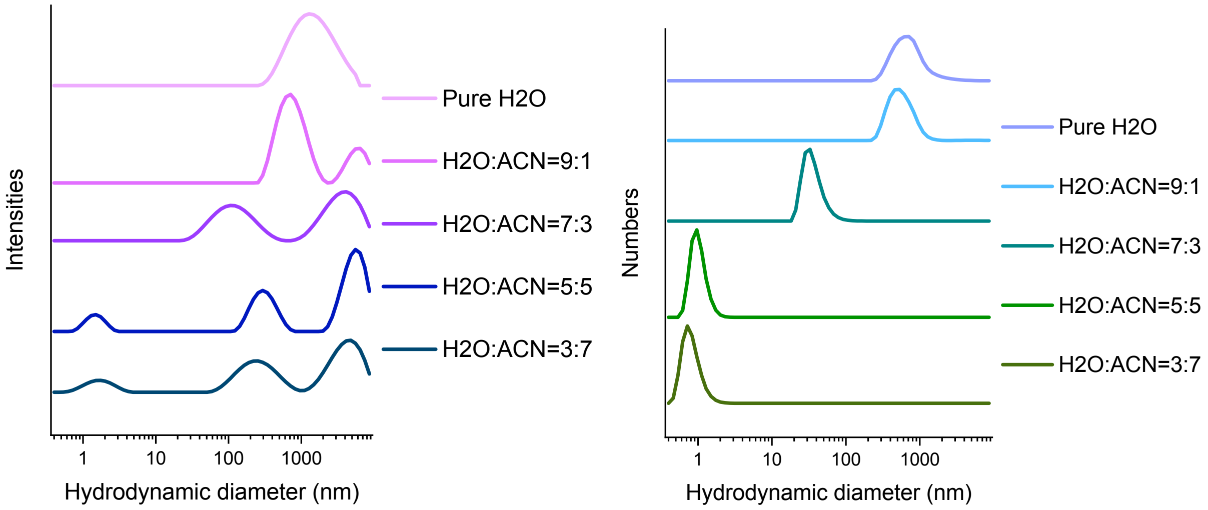

Figure S20. DLS of the **Ir-COOH** complex dissolved in acetonitrile/water mixtures used for the photoluminescence experiments reported in Figure S18: Size distribution by intensities (left) and size distribution by numbers (right).

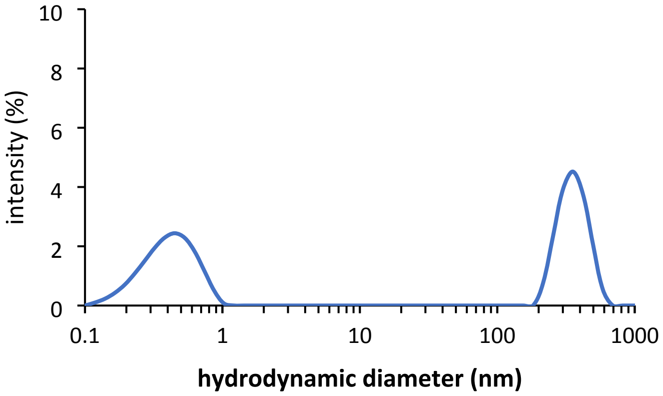

Figure S21. DLS of the **Ir-COOH** complex dissolved in methanol at the same concentration used for the photoluminescence experiments.

VI. Photochemical stability of Ir-COOH and Ir-PNA

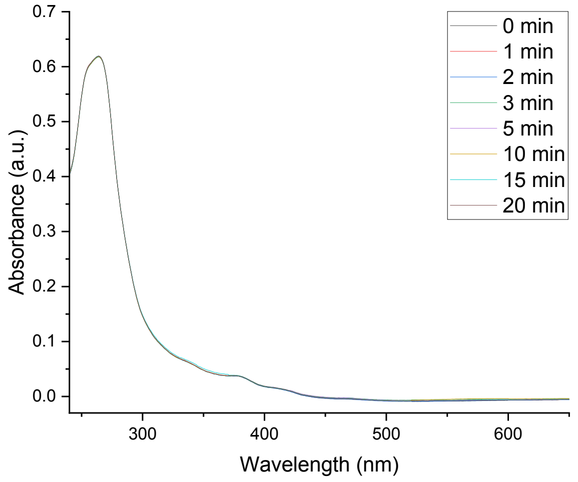

Figure S22. UV-Vis absorption spectra of a CH_2Cl_2/methanol (9/1) solution of the **Ir-PNA** presaturated with O_2, at different irradiation times.

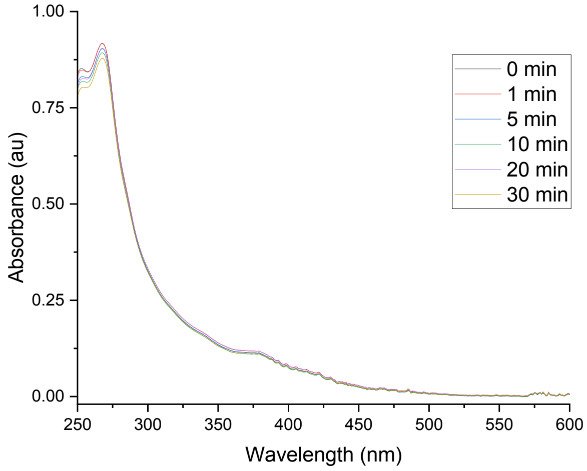

Figure S23. UV-Vis absorption spectra of a methanol solution of the **Ir-COOH** presaturated with O_2, at different irradiation times. The irradiation was carried out with a UV lamp (semi-permanent nail polish lamp) with a total power of 5.2 mW/cm^2 measured from 200-800 nm.

VII. UV-LED and UV-lamp emissions

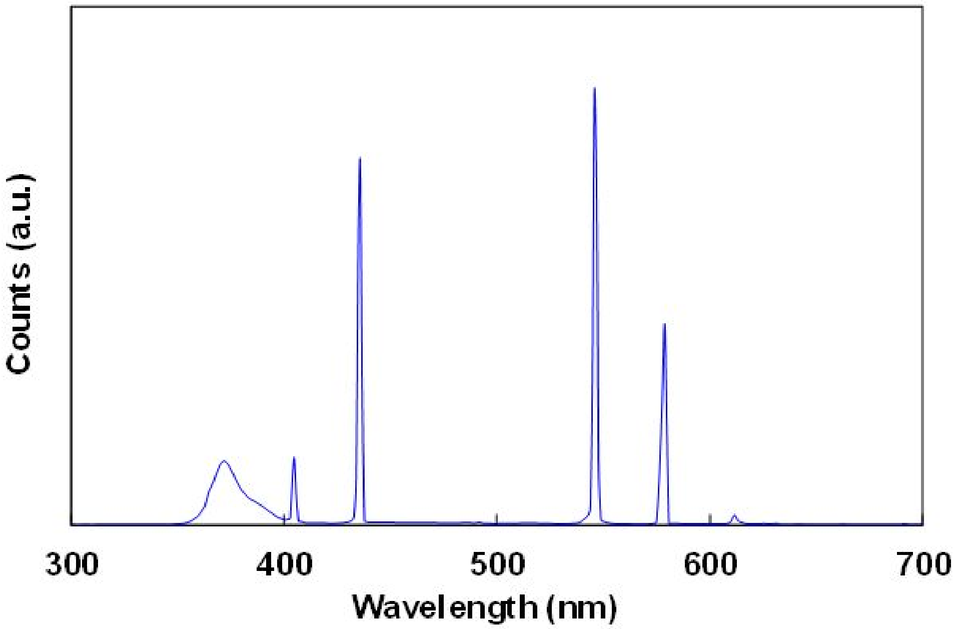

Figure S24. Emission profile of the UV lamp (semi-permanent nail polish lamp) used in the phototoxicity tests on HeLa cells.

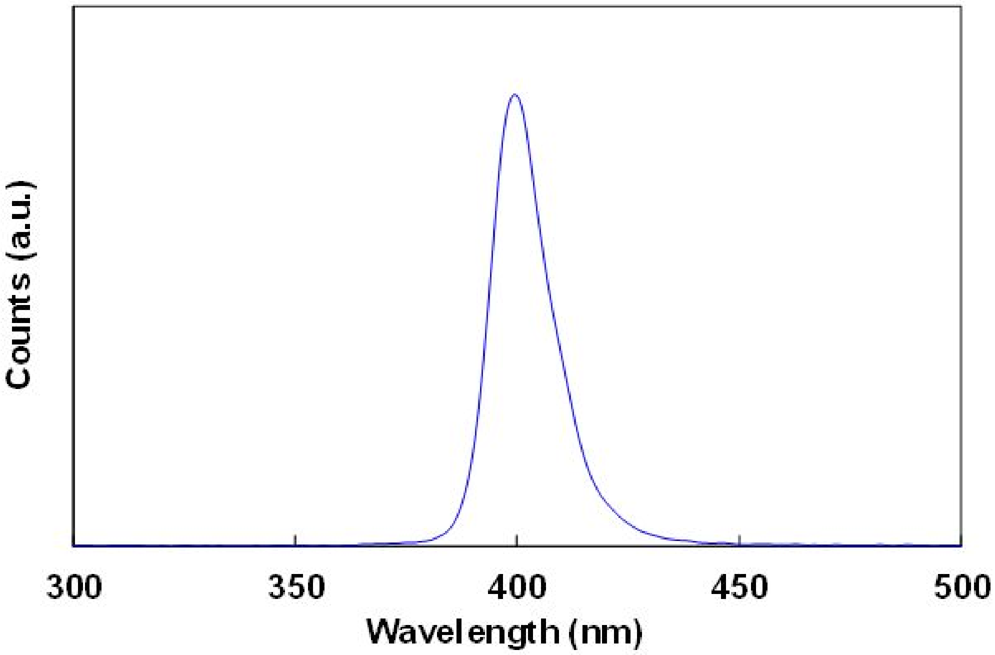

Figure S25. Emission profile of the UV-LED.
